# Transient Elastography in Alcoholic Liver Disease and Nonalcoholic Fatty Liver Disease: A Systemic Review and Meta-Analysis

**DOI:** 10.1155/2021/8859338

**Published:** 2021-01-20

**Authors:** Changzhou Cai, Xin Song, Xueyang Chen, Weihua Zhou, Qi Jin, Shenghui Chen, Feng Ji

**Affiliations:** ^1^Department of Gastroenterology, The First Affiliated Hospital of Zhejiang University School of Medicine, Hangzhou 310003, China; ^2^Department of Gastroenterology, Sanmen People's Hospital, Taizhou, China

## Abstract

**Background and Aims:**

Alcoholic liver disease (ALD) and nonalcoholic fatty liver disease (NAFLD) have become common chronic liver diseases. Recent evidence has shown the value of transient elastography (TE) in the context of ALD/NAFLD. The aim of this study is to investigate the accuracy of TE for diagnosing steatosis and fibrosis in ALD/NAFLD patients.

**Methods:**

We retrieved relevant English studies from the databases of PubMed, Embase, the Web of Science, and the Cochrane Library through March 31^st^ 2019. We included studies regarding the diagnosis or staging of steatosis or fibrosis by using controlled attenuation parameter (CAP) or liver stiffness measurement (LSM) measured by TE in patients with ALD or NAFLD. The reference standard of all included studies was liver biopsy. A random-effects model was applied. Statistical analyses were performed using STATA.

**Results:**

A total of 62 articles were included and analyzed in our meta-analysis. In patients with ALD/NAFLD, the pooled results revealed that the sensitivity and specificity of CAP were 0.84, 0.83, and 0.78 and 0.83, 0.71, and 0.62 for steatosis grades **≥***S*1, **≥**S2, and =*S*3, respectively. The sensitivity and specificity of LSM for identifying fibrosis grades **≥***F*1, **≥**F2, **≥**F3, and =*F*4 were 0.77, 0.77, 0.83, and 0.91 and 0.80, 0.82, 0.84, and 0.86, respectively.

**Conclusion:**

In patients with ALD/NAFLD, CAP was feasible for identifying and screening steatosis, and LSM was accurate for diagnosing fibrosis, especially severe fibrosis and cirrhosis.

## 1. Introduction

Nonalcoholic fatty liver disease (NAFLD) is one of the most prevalent chronic liver diseases worldwide, affecting approximately 25% of the adult population [1]. It is expected that NAFLD will soon become the leading cause of liver transplantation [2, 3]. The spectrum of NAFLD ranges from the reversible phase of nonalcoholic fatty liver disease (NAFLD) to nonalcoholic steatohepatitis (NASH), which may progress to liver fibrosis and hepatocellular carcinoma [4]. Alcoholic liver disease (ALD) is another common chronic liver disease that includes the steatosis and fibrosis pathological classifications [5]. Currently, liver biopsy is still the gold standard for ALD/NAFLD diagnosis and steatosis and liver fibrosis staging. However, the application of liver biopsy is not easy because it is an invasive test with potentially life-threatening complications after the operation [6]. Therefore, developing noninvasive and accurate methods for disease diagnosis and staging in ALD/NAFLD patients is urgently needed.

Recently, controlled attenuation parameter (CAP) and liver stiffness measurement (LSM) measured by transient elastography (TE) with Fibroscan^®^ equipment (Echosens, Paris, France) have been widely used to assess hepatic steatosis and liver fibrosis, respectively [7, 8]. CAP is evaluated based on the properties of ultrasonic signals acquired by the Fibroscan^®^ equipment. Moreover, CAP and LSM shared the same radio-frequency data and the same region of interest [9]. Many biopsy-proven ALD/NAFLD studies have reported excellent performance of CAP in detecting and staging steatosis [10–12]. Additionally, LSM accurately predicted liver fibrosis and differentiated its different stages in several biopsy-controlled studies [9, 13, 14].

In this meta-analysis, we aimed to evaluate the efficiency of CAP and LSM measured by TE for diagnosing and staging steatosis and fibrosis in patients with ALD/NAFLD using liver biopsy as the reference standard.

## 2. Methods and Materials

### 2.1. Search Strategy

We performed this comprehensive systematic review and meta-analysis based on the Preferred Reporting Items for Systematic Reviews and Meta-Analyses (PRISMA) statement. Studies published in English about the diagnosis and staging of steatosis and fibrosis using CAP and LSM in patients with ALD/NAFLD were retrieved from the PubMed, Embase, Web of Science, and Cochrane Library databases. The databases were searched through December 31^st^, 2019. The electronic search strategy included the following terms: (“alcoholic liver disease” OR “ALD” OR “alcoholic hepatitis” OR “non-alcoholic fatty liver disease” OR “NAFLD” OR “non-alcoholic steatohepatitis” OR “NASH”) AND (“transient elastography” OR “controlled attenuation parameter” OR “liver stiffness”). The retrieval strategies for each database are shown in the supplementary materials. Furthermore, we performed a manual search, and we added two additional suitable articles.

### 2.2. Paper Selection

We included studies if they met the following criteria: (1) studies regarding the diagnosis or staging of steatosis or fibrosis by using CAP or LSM measured by TE in patients with ALD or NAFLD; (2) studies in which the gold standard diagnosis method for patients with NAFLD/ALD was liver biopsy; and (3) articles that provided specificity (SPE), sensitivity (SEN), sample size, or enough information to calculate and construct a diagnostic 2 ∗ 2 contingency table. The exclusion criteria included the following: (1) cell or animal studies, comments, reviews, and letters; (2) duplicate studies; and (3) studies focused on irrelevant topics or that did not report necessary data.

## 3. Methodological Quality and Bias Assessment

The methodological quality of the included studies was evaluated by the Quality Assessment of Diagnostic Accuracy Studies-2 (QUADAS-2) checklist by Review Manager Version 5.3. This evaluation form consists of four parts: patient selection, index test, reference standard, and flow and timing. We assessed the methodological quality of each article by answering every question using “Yes,” “No,” or “Unclear” or “High concern,” “Low concern,” or “Unclear concern.”

### 3.1. Data Extraction

Two researchers (CC and XC) independently filtered studies by reviewing the titles and abstracts and then proceeded with a full-text evaluation. The third reviewer resolved the disagreements. We extracted the following data from the literature: (1) author, publication year, ethnicity, age, sample size, disease, diagnostic index, probe type, diagnostic thresholds (cut-off values), body mass index (BMI), and study design; (2) diagnostic parameters of the diagnostic index, including sensitivity, specificity, and calculated or extracted numbers of true positive (TP), false positive (FP), false negative (FN), and true negative (TN) cases. We then constructed a diagnostic 2 ∗ 2 contingency table.

### 3.2. Statistical Analysis

Our meta-analysis was mainly divided into two parts. First, we assessed TE-measured CAP for the diagnosis and grading of steatosis in patients with ALD/NAFLD. Subgroup analyses were then conducted based on BMI and cut-off value when analyzing the efficiency of CAP for grade ≥*S*1, ≥S2, and S3 steatosis. Meta-regression analyses were performed to determine the source of heterogeneity. Second, we evaluated TE-measured LSM for the diagnosis and grading of fibrosis in patients with ALD/NAFLD. When analyzing the efficiency of LSM for grade ≥*F*1, ≥F2, ≥F3, and F4 fibrosis, we performed subgroup analyses based on disease status, BMI, and study design. Meta-regression analyses were performed to determine the source of heterogeneity. We estimated SEN, SPE, positive likelihood ratio (PLR), negative likelihood ratio (NLR), and diagnostic odds ratio (DOR) with their associated 95% confidence intervals (CIs) from the diagnostic 2 ∗ 2 contingency tables. To analyze the diagnostic accuracy of TE in patients with ALD/NAFLD, we pooled SEN and SPE graphically using forest plots, constructed summary receiver-operating characteristics (SROC) curves, and calculated areas under the SROC curve (AUC).

We evaluated the heterogeneity between studies using the I^2^ index. I^2^ values of 25%, 50%, and 75% corresponded to low, medium, and high heterogeneity, respectively. The random-effects model was chosen as the default; if the I^2^ values were less than 50%, we chose the fix-effects model. Furthermore, we constructed the Deeks' funnel plots, in which *P* < 0.10 indicated that there was publication bias. We performed all of these statistical analyses using Stata (version 12.0).

## 4. Results

### 4.1. Paper Selection and Characteristics

**Figure 1:**
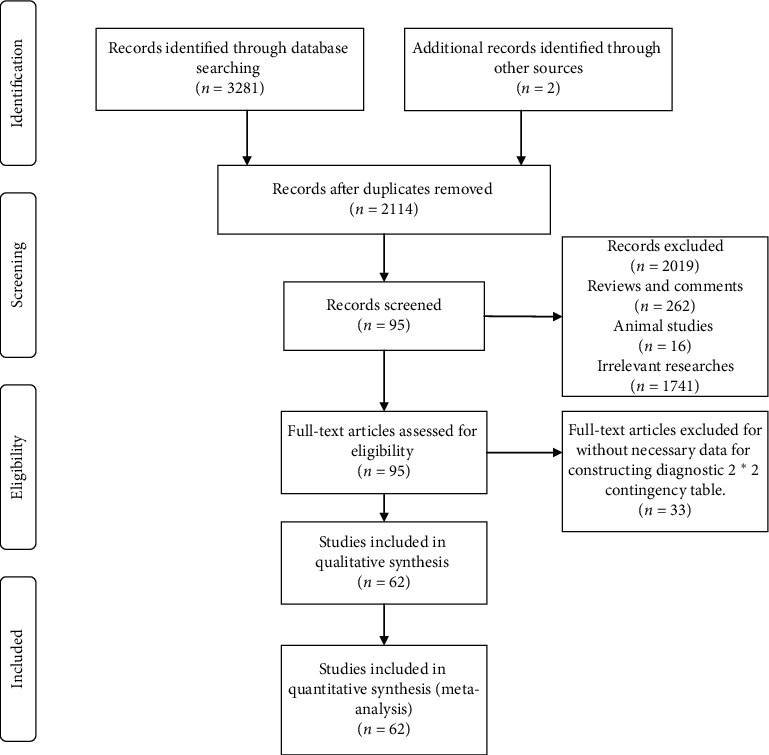
Flowchart showing the selection of articles included in the meta-analysis.

Based on the literature research strategy, we retrieved 3283 articles, of which 469 were from PubMed, 1491 were from Embase, 1313 were from the Web of Science, eight were from Cochrane, and two were identified in the manual search. After removing 1069 duplicate studies, 262 reviews or comments, 16 animal studies, 1741 irrelevant studies, and 33 studies failing to provide sufficient data, we eventually included 62 articles (supplementary materials) in our study (Figure 1). The necessary information and related diagnostic data are shown in Table S1. Among the studies, 17 assessed the efficiency of CAP for diagnosing and staging steatosis in ALD/NAFLD patients, and 53 studies evaluated the accuracy of LSM for diagnosing and grading fibrosis in ALD/NAFLD patients. CAP and LSM were both measured by TE with Fibroscan^®^ equipment. The methodological quality of each included article assessed by QUADAS is shown in a bar chart (Figure S1).

### 4.2. Diagnostic Performance of CAP for Steatosis in ALD/NAFLD Patients

**Figure 2:**
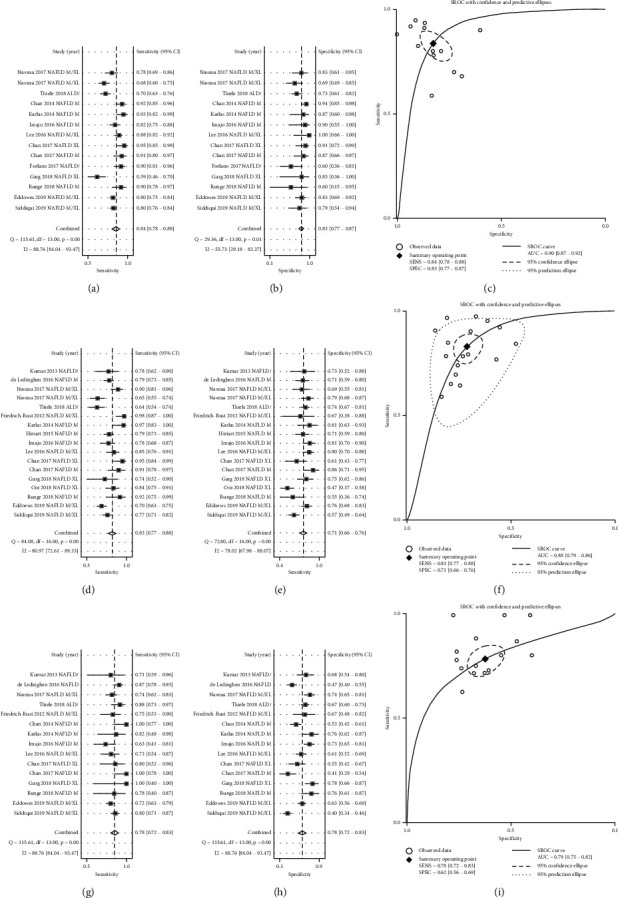
Diagnostic performance of controlled attenuation parameter (CAP) for steatosis in alcoholic liver disease/nonalcoholic fatty liver disease. (a) Sensitivity, (b) specificity, and (c) summary receiver-operating characteristics (SROC) curve of CAP for identifying patients with steatosis grade ≥*S*1. (d) Sensitivity, (e) specificity, and (f) SROC curve of CAP for identifying patients with steatosis grade ≥*S*2. (g) Sensitivity, (h) specificity, and (i) SROC curve of CAP for identifying patients with steatosis grade =*S*3. CAP: controlled attenuation parameter.

14 studies containing 1936 ALD/NAFLD patients with pathologically confirmed S1–S3 steatosis and 369 healthy controls assessed the diagnostic accuracy of CAP for steatosis grades ≥*S*1. The pooled results indicated high accuracy with an AUC of 0.90 (95% CI 0.87–0.92) (Figure 2(c)), a sensitivity of 0.84 (95% CI 0.78–0.88) (Figure 2(a)), and a specificity of 0.83 (95% CI 0.77–0.87) (Figure 2(b)) at an average threshold of 272 dB/m. Publication bias was not significant (*P*=0.462) (Figure S2).

17 studies containing 1625 pathologically confirmed ALD/NAFLD patients with S2–S3 steatosis grades and 1542 controls assessed the diagnostic accuracy of CAP for steatosis grades ≥*S*2. The pooled results revealed high accuracy with an AUC of 0.83 (95% CI 0.79–0.86) (Figure 2(f)), sensitivity of 0.83 (95% CI 0.77–0.88) (Figure 2(d)), and specificity of 0.71 (95% CI 0.66–0.76) (Figure 2(e)) at an average threshold of 292 dB/m. There was significant publication bias, with *P* < 0.05 (Figure S3).

15 studies containing 591 patients with pathologically confirmed ALD/NAFLD with S2–S3 steatosis and 1872 controls assessed the diagnostic accuracy of CAP for steatosis grade S3. Accuracy was lowest at the S3 threshold compared with the ≥*S*1 threshold and the ≥*S*2 threshold, with an AUC of 0.79 (95% CI 0.75–0.82) (Figure 2(i)), sensitivity of 0.78 (95% CI 0.72–0.83) (Figure 2(g)), and specificity of 0.62 (95% CI 0.56–0.69) (Figure 2(h)) at an average threshold of 308 dB/m. The results of PLR, NLR, and DOR from the nomogram are shown in supplementary materials (Figures S2–S4).

We then performed subgroup analyses according to BMI and the cut-off values (Table 1). Furthermore, meta-regression was performed according to covariates including BMI, cut-off value, sample size, ethnicity, disease, and study design, and disease was shown to relate to the heterogeneity (*P*=0.045) (Table S3–S5).

### 4.3. Diagnostic Performance of LSM for Fibrosis in ALD/NAFLD Patients

**Figure 3:**
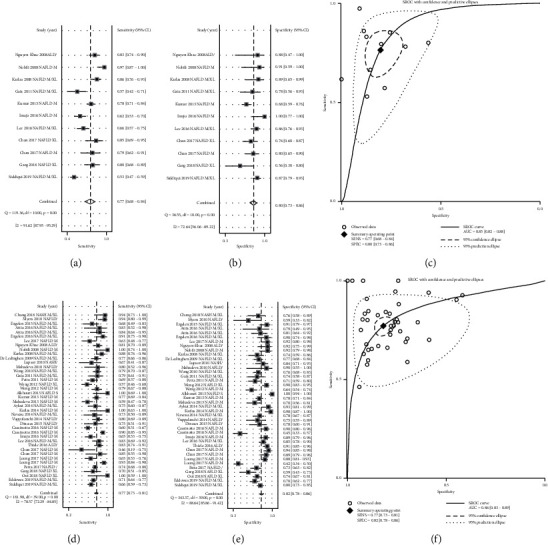
Diagnostic performance of LSM for liver fibrosis in alcoholic liver disease/nonalcoholic fatty liver disease. (a) Sensitivity, (b) specificity, and (c) summary receiver-operating characteristics (SROC) curve of LSM for identifying patients with fibrosis grade ≥*F*1. (d) Sensitivity, (e) specificity, and (f) SROC curve of LSM for identifying patients with fibrosis grade ≥*F*2. LSM: liver stiffness measurement; SROC: summary receiver-operating characteristics.

11 studies containing 1097 patients with pathologically confirmed ALD/NAFLD with F1–F4 fibrosis grades and 484 healthy controls assessed the diagnostic performance of LSM for fibrosis grades ≥*F*1. Accuracy was lowest at the ≥*F*1 threshold, with an AUC of 0.85 (95% CI 0.82–0.88) (Figure 3(c)), a sensitivity of 0.77 (95% CI 0.68–0.74) (Figure 3(a)), and a specificity of 0.80 (95% CI 0.73–0.86) (Figure 3(b)) at an average threshold of 6.3 kPa. Significant publication bias was identified (*P*=0.029) in this analysis (Figure S5).

40 studies containing 2569 ALD/NAFLD patients with pathologically confirmed F2–F4 fibrosis and 3014 controls assessed the diagnostic performance of LSM for fibrosis grades ≥*F*2. The pooled results showed high accuracy with an AUC of 0.86 (95% CI 0.83–0.89) (Figure 3(f)), sensitivity of 0.77 (95% CI 0.73–0.81) (Figure 3(d)), and specificity of 0.82 (95% CI 0.78–0.86) (Figure 3(e)) at an average threshold of 8.2 kPa. We also identified significant publication bias, with *P* < 0.05 (Figure S6).

**Figure 4:**
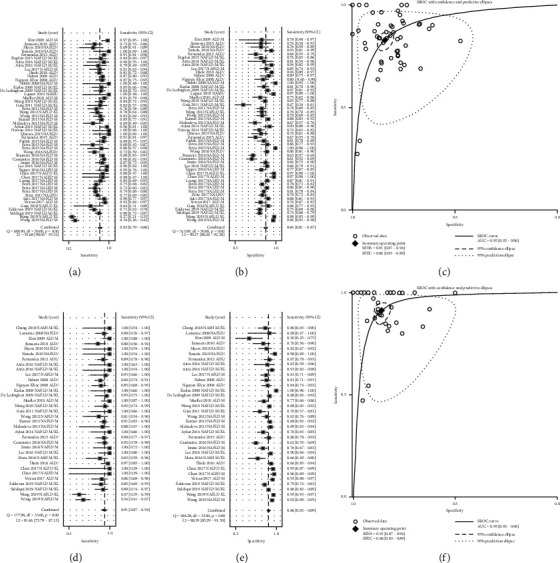
Diagnostic performance of liver stiffness measurement (LSM) for liver fibrosis in alcoholic liver disease/nonalcoholic fatty liver disease. (a) Sensitivity, (b) specificity, and (c) summary receiver-operating characteristics (SROC) curve of LSM for identifying patients with fibrosis grade ≥*F*3. (d) Sensitivity, (e) specificity, and (f) SROC curve of LSM for identifying patients with fibrosis grade=*F*4. LSM: liver stiffness measurement; SROC: summary receiver-operating characteristic.

51 studies containing 2925 patients pathologically confirmed ALD/NAFLD with F3–F4 fibrosis and 6308 controls assessed the diagnostic performance of LSM for fibrosis grades ≥*F*3. Diagnostic accuracy was high at the ≥*F*3 threshold, with an AUC of 0.90 (95% CI 0.88–0.93) (Figure 4(c)), sensitivity of 0.83 (95% CI 0.79–0.86) (Figure 4(a)), and specificity of 0.84 (95% CI 0.81–0.87) (Figure 4(b)) at an average threshold of 13.4 kPa. Publication bias was also detected, with *P* < 0.05 (Figure S7).

34 studies containing 914 ALD/NAFLD patients with pathologically confirmed F4 fibrosis and 4238 controls assessed the diagnostic performance of LSM for fibrosis grade F4. Diagnostic accuracy was highest at the F4 threshold, with an AUC of 0.95 (95% CI 0.92–0.96) (Figure 4(f)), sensitivity of 0.91 (95% CI 0.87–0.94) (Figure 4(d)), and specificity of 0.86 (95% CI 0.83–0.89) (Figure 4(e)) at an average threshold of 14.2 kPa. No significant publication bias was detected (*P*=0.054) (Figure S8). The results of PLR, NLR, and DOR from the nomogram are shown in supplementary materials (Figures S5–S7).

In addition, subgroup analyses were conducted according to BMI and study type (Table 2). We then conducted meta-regressions according to the sample size, ethnicity, disease, and study design, and we detected that disease was the possible source of heterogeneity, with *P*=0.042 (Table S6–S9).

## 5. Discussion

ALD and NAFLD are two common chronic liver diseases that pathologically range from steatosis, steatohepatitis, and fibrosis to cirrhosis [7, 15]. Thus far, liver biopsy is still the gold standard for diagnosing patients with ALD/NAFLD and staging steatosis and fibrosis. However, the application of liver biopsy is difficult due to its invasive characteristics, possible subsequent adverse reactions, and relatively high price. Therefore, a variety of noninvasive methods, including serum biomarkers and imaging techniques, have been evolving and advancing [16, 17]. When compared with traditional noninvasive diagnostic methods for ALD/NAFLD in clinical practice, such as ultrasound and computed tomography scans, TE using Fibroscan^®^ equipment exhibited high accuracy in diagnosing and staging steatosis and fibrosis for patients with ALD/NAFLD [7, 18–20]. Considering the pathological commonalities between ALD and NAFLD, this meta-analysis focused on the population of ALD and NAFLD patients and consisted of two parts. First, we studied the accuracy of TE-measured CAP in the diagnosis and grading of steatosis in patients with ALD/NAFLD. We found that the average cut-off values of CAP for identifying patients with steatosis grades **≥***S*1, **≥**S2, and =*S*3 were 272 dB/m, 292 dB/m, and 308 dB/m, respectively. Previous studies obtained different results regarding CAP measuring the steatosis grades of ALD/NAFLD patients. Early research showed the excellent diagnostic performance of CAP in steatosis, with AUCs of 0.91, 0.95, and 0.89 for steatosis grades **≥***S*1, **≥**S2, and =*S*3, respectively [21]. Subsequent research failed to repeat such excellent diagnostic efficiency, and most studies showed high diagnostic accuracy for steatosis grades ≥*S*1, while a decline in AUCs was observed in the diagnostic performance for steatosis grades ≥*S*2 and =*S*3 [12, 22–24]. In our meta-analysis, the diagnostic accuracy of CAP decreased as the steatosis grades increased, with the highest AUC of 0.90 obtained for steatosis grades **≥ ***S*1, but the AUCs were 0.83 for steatosis grades **≥***S*2 and 0.79 for steatosis grade S3. The pooled results revealed that the sensitivity and specificity of CAP were 0.84, 0.83, and 0.78 and 0.83, 0.71, and 0.62 for steatosis grades **≥ ***S*1, **≥**S2, and =*S*3, respectively. The reason for the reduction in the accuracy of CAP in grading steatosis as the steatosis deteriorated is still unclear but might relate to the characteristics of the study population, especially the ethnicity, BMI, and proportion of patients with severe steatosis [25]. Further subgroup analyses indicated that CAP exhibited higher diagnostic accuracy in studies whose population BMI was <28 kg/m^2^ than in studies whose population BMI was **≥**28 kg/m^2^. CAP in the studies with thresholds <270 dB/m, <290 dB/m, and <300 dB/m had higher accuracy in the diagnosis of steatosis grades **≥***S*1, **≥**S2, and =S3, respectively, than that in the study with threshold **≥**270 dB/m, **≥**290 dB/m, and **≥**300 dB/m. This finding might be associated with the relatively higher BMI of the population in studies with higher CAP cut-off values because higher BMI leads to less satisfactory results. The disease was the only possible factor related to the heterogeneity when exploring the diagnostic performance of CAP for steatosis grades ≥*S*1 in ALD/NAFLD patients, and separate analyses showed higher diagnostic accuracy of CAP in NAFLD patients than in ALD patients (sensitivity 0.85 vs. 0.70, specificity 0.84 vs. 0.73, and AUC 0.91 vs. 0.77).

Second, we analyzed the efficiency of TE-measured LSM in the diagnosis and grading of liver fibrosis in patients with ALD/NAFLD. The average cut-offs for LSM were 6.3 kPa, 8.2 kPa, 13.4 kPa, and 14.2 kPa for patients with fibrosis grades **≥***F*1, **≥**F2, **≥**F3, and =*F*4, respectively. Previous studies have shown that TE exhibited a high degree of accuracy in the diagnosis of liver fibrosis by measuring LSM, especially in cases of severe fibrosis and cirrhosis [12, 25–27]. Consistent with these results, our pooled data demonstrated that the AUCs of LSM for diagnosing fibrosis grades **≥***F*1, **≥**F2, **≥**F3, and =*F*4 were 0.85, 0.86, 0.90, and 0.95, respectively. The sensitivity and specificity of LSM for identifying fibrosis grades **≥ ***F*1, **≥**F2, **≥**F3, and =*F*4 were 0.77, 0.77, 0.83, and 0.91 and 0.80, 0.82, 0.84, and 0.86, respectively. Subsequent subgroup analyses revealed that patients with BMI <28 kg/m^2^ were more sensitive to TE than patients with BMI **≥**28 kg/m^2^, with the data showing that TE had higher accuracy in the lower BMI population than in the higher BMI population. Meta-regression analyses identified that disease might be the reason for the heterogeneity when studying the diagnostic performance of LSM for fibrosis grades ≥*F*3 in ALD/NAFLD patients, but subgroup analyses showed that there was no obvious difference in TE diagnostic accuracy between analyses with ALD patients and NAFLD patients (sensitivity 0.81 vs. 0.80, specificity 0.85 vs. 0.89, and AUC 0.90 vs. 0.92).

Although we are trying to comprehensively analyze the value of TE in the context of ALD/NAFLD, there are still many weaknesses in this meta-analysis. First, the CAP and LSM of all the studies we included had different thresholds. Although we calculated the average cut-offs of CAP for the diagnosis of every steatosis grade and the average cut-offs of LSM for the determination of every fibrosis grade, we could not obtain the best cut-offs for CAP and LSM for diagnosis. Second, we conducted a comprehensive electronic search and manual search of multiple databases. However, in the subsections of our study, significant publication bias was detected, which indicated that some failed or poorly performed research was missed or not included in SCI journals. Third, in the analysis process, many analyses had significant heterogeneity, and meta-regression could not find all sources of heterogeneity. The pooled results were relatively more reliable after excluding the effects of a single factor by subgroup analysis. In general, the existing heterogeneity does not affect our interpretation of the results. Despite these shortcomings in this study, we can still draw some very valuable and strong conclusions from this analysis: (1) this is the first meta-analysis to comprehensively study the diagnostic accuracy of CAP and LSM for steatosis and fibrosis in ALD/NAFLD patients; (2) the accuracy of TE used for diagnosing steatosis or fibrosis between ALD and NAFLD patients was equivalent; (3) when assessing the accuracy of CAP to diagnose different grades of steatosis in ALD/NAFLD patients, we found that CAP measured by TE was good at screening for patients with fatty liver but failed to maintain high accuracy in diagnosing patient with severe steatosis; (4) when evaluating the accuracy of LSM to diagnose different grades of fibrosis in ALD/NAFLD patients, we found that TE was accurate for diagnosing fibrosis, especially severe fibrosis and cirrhosis; and (5) because of the large number of studies and participants in our meta-analysis, the results are convincing and can guide clinical practice.

In conclusion, this study comprehensively analyzed the applicability of TE for diagnosing steatosis and fibrosis in ALD/NAFLD patients. The pooled results indicated that TE exhibited similar diagnostic efficiency for ALD and NAFLD patients. In patients with ALD/NAFLD, CAP was feasible for identifying steatosis, and LSM was accurate for diagnosing fibrosis, especially severe fibrosis and cirrhosis.

## Figures and Tables

**Table 1:** Summary diagnostic accuracy parameter estimates and their 95% confidence intervals.

Subgroup		Sensitivity (95% CI)	Specificity (95% CI)	Positive LR (95% CI)	Negative LR (95% CI)	DOR (95% CI)	AUC
S1–S3							
BMI ≥28	6	0.76 [0.69–0.81]	0.79 [0.70–0.86]	3.69 [2.41–5.66]	0.30 [0.23–0.41]	12.11 [6.08–24.12]	0.85 [0.81–0.88]
BMI <28	7	0.89 [0.83–0.93]	0.88 [0.80–0.93]	7.44 [4.24–13.06]	0.13 [0.07–0.21]	59.05 [22.16–159.76]	0.95 [0.92–0.96]
Cut-off ≥270	7	0.77 [0.73–0.81]	0.82 [0.78–0.86]	4.34 [3.45–5.46]	0.28 [0.23–0.33]	15.65 [11.33–21.60]	0.85 [0.83–0.89]
Cut-off <270	7	0.89 [0.85–0.92]	0.86 [0.74–0.89]	6.56 [3.20–13.47]	0.13 [0.10–0.17]	50.89 [21.26–121.84]	0.92 [0.89–0.94]
S2–S3							
BMI ≥28	10	0.79 [0.73–0.84]	0.69 [0.62–0.75]	2.56 [2.09–3.13]	0.30 [0.23–0.38]	8.61 [6.07–2.21]	0.81 [0.77–0.84]
BMI <28	7	0.88 [0.78–0.94]	0.88 [0.78–0.90]	3.35 [2.60–4.31]	0.16 [0.09–0.31]	38.28 [25.84–56.70]	0.84 [0.80–0.87]
Cut-off ≥290	9	0.76 [0.70–0.82]	0.70 [0.64–0.75]	2.52 [2.15–2.95]	0.34 [0.27–0.42]	7.45 [5.56–9.97]	0.79 [0.75–0.82]
Cut-off <290	8	0.89 [0.75–0.81]	0.73 [0.63–0.81]	3.30 [2.33–4.65]	0.15 [0.09–0.25]	31.19 [5.23–17.08]	0.89 [0.86–0.92]
=S3							
BMI ≥28	7	0.75 [0.69–0.80]	0.63 [0.52–0.73]	2.03 [1.60–2.58]	0.40 [0.33–0.47]	5.13 [3.62–7.26]	0.77 [0.73–0.80]
BMI <28	8	0.86 [0.74–0.93]	0.62 [0.54–0.69]	2.25 [1.90–2.67]	0.23 [0.13–0.42]	9.75 [5.05–18.81]	0.78 [0.74–0.81]
Cut-off ≥300	11	0.75 [0.70–0.79]	0.64 [0.56–0.71]	2.10 [1.74–2.54]	0.39 [0.33–0.46]	5.40 [3.98–7.32]	0.77 [0.73–0.80]
Cut-off <300	4	0.91 [0.85–0.95]	0.83 [0.77–0.87]	2.31 [1.84–2.89]	0.06 [0.01–0.73]	37.38 [3.29–424.09]	0.94 [0.92–0.96]

LR: likelihood ratio, DOR: diagnostic odds ratio, AUC: area under the curve, and CI: confidence interval.

**Table 2:** Summary diagnostic accuracy parameter estimates and their 95% confidence intervals.

Subgroup		Sensitivity (95% CI)	Specificity (95% CI)	Positive LR (95% CI)	Negative LR (95% CI)	DOR (95% CI)	AUC
F2–F4							
BMI ≥28 kg/m^2^	21	0.74 [0.69–0.78]	0.78 [0.73–0.83]	3.42 [2.80–4.18]	0.33 [0.29–0.39]	10.24 [7.94–13.21]	0.82 [0.79–0.85]
BMI <28 kg/m^2^	14	0.80 [0.72–0.86]	0.85 [0.77–0.90]	5.26 [3.56–7.79]	0.24 [0.18–0.32]	22.06 [13.64–35.69]	0.89 [0.86–0.91]
Prospective	37	0.77 [0.73–0.81]	0.82 [0.78–0.86]	4.34 [3.45–5.46]	0.28 [0.23–0.33]	15.65 [11.33–21.60]	0.86 [0.83–0.89]
Retrospective	3	0.74 [0.65–0.81]	0.82 [0.73–0.88]	3.51 [1.94–6.34]	0.31 [0.18–0.54]	14.74 [7.41–29.32]	0.87 [0.83–0.90]
F3–F4							
NAFLD	42	0.81 [0.77–0.85]	0.85 [0.82–0.88]	10.09 [2.16–47.13]	0.14 [0.04–0.53]	25.19 [18.15–34.95]	0.90 [0.87–0.92]
ALD	9	0.80 [0.72–0.86]	0.89 [0.85–0.92]	5.53 [4.44–6.88]	0.22 [0.18–0.27]	31.19 [5.23–17.08]	0.92 [0.89–0.94]
BMI ≥28 kg/m^2^	25	0.80 [0.73–0.85]	0.84 [0.79–0.88]	5.00 [3.91–6.40]	0.24 [0.18–0.32]	20.57 [14.59–29.00]	0.89 [0.86–0.91]
BMI <28 kg/m^2^	23	0.86 [0.83–0.89]	0.86 [0.82–0.90]	6.26 [4.67–8.37]	0.16 [0.13–0.20]	38.28 [25.84–56.70]	0.92 [0.89–0.94]
Prospective	43	0.82 [0.78–0.86]	0.84 [0.81–0.88]	5.30 [4.34–6.48]	0.21 [0.17–0.26]	25.20 [18.68–39.99]	0.90 [0.87–0.93]
Retrospective	8	0.80 [0.72–0.86]	0.89 [0.85–0.92]	5.53 [4.44–6.88]	0.22 [0.18–0.27]	31.19 [5.23–17.08]	0.92 [0.89–0.94]
=F4							
NAFLD	25	0.92 [0.86–0.96]	0.87 [0.83–0.90]	7.18 [5.44–9.47]	0.09 [0.05–0.17]	78.59 [39.17–157.70]	0.95 [0.93–0.97]
ALD	9	0.92 [0.84–0.96]	0.84 [0.76–0.89]	5.64 [3.83–8.30]	0.10 [0.05–0.20]	57.52 [26.27–125.92]	0.94 [0.92–0.96]
BMI ≥28 kg/m^2^	12	0.88 [0.78–0.94]	0.85 [0.80–0.89]	5.91 [4.51–7.75]	0.14 [0.07–0.26]	43.04 [23.05–80.35]	0.92 [0.90–0.94]
BMI <28 kg/m^2^	17	0.92 [0.86–0.95]	0.88 [0.82–0.92]	7.60 [4.97–11.61]	0.09 [0.06–0.16]	80.28 [38.22–168.63]	0.95 [0.93–0.97]
Prospective	29	0.92 [0.86–0.95]	0.87 [0.83–0.90]	6.90 [5.36–8.88]	0.10 [0.06–0.16]	71.57 [39.82–128.65]	0.95 [0.93–0.96]
Retrospective	5	0.91 [0.85–0.95]	0.83 [0.770.87]	5.29 [3.90–7.17]	0.10 [0.06–0.19]	51.00 [24.71–105.24]	0.94 [0.92–0.96]

LR: likelihood ratio, DOR: diagnostic odds ratio, AUC: area under the curve, and CI: confidence interval.

## Data Availability

All data of this study are available from the corresponding author upon request by email.

## Supplementary Materials

Supplementary Materials Table S1: characteristics of the included studies. Table S2: the meta-regression analysis of CAP detecting patients with steatosis grade ≥*S*1. Table S3: the meta-regression analysis of CAP detecting patients with steatosis grade ≥*S*2. Table S4: the meta-regression analysis of CAP detecting patients with steatosis grade =*S*3. Table S5: the meta-regression analysis of CAP detecting patients with fibrosis grade ≥*F*1. Table S6: the meta-regression analysis of CAP detecting patients with fibrosis grade ≥*F*2. Table S7: the meta-regression analysis of CAP detecting patients with fibrosis grade ≥*F*3. Table S8: the meta-regression analysis of CAP detecting patients with fibrosis grade =*F*4. Figure S1: overall methodology quality assessment of included articles using the QUADAS-2 criteria. Figure S2: diagnostic performance of CAP for steatosis grade ≥*S*1 in alcoholic liver disease/nonalcoholic fatty liver disease. A: positive likelihood; B: negative likelihood; C: diagnostic odds ratio; D: Deeks' funnel plots; E: Fagan's nomogram. Figure S3: diagnostic performance of CAP for steatosis grade ≥*S*2 in alcoholic liver disease/nonalcoholic fatty liver disease. A: positive likelihood; B: negative likelihood; C: diagnostic odds ratio; D: Deeks' funnel plots; E: Fagan's nomogram. Figure S4: diagnostic performance of CAP for steatosis grade =*S*3 in alcoholic liver disease/nonalcoholic fatty liver disease. A: positive likelihood; B: negative likelihood; C: diagnostic odds ratio; D: Deeks' funnel plots; E: Fagan's nomogram. Figure S5: diagnostic performance of LSM for fibrosis grade ≥*F*1 in alcoholic liver disease/nonalcoholic fatty liver disease. A: positive likelihood; B: negative likelihood; C: diagnostic odds ratio; D: Deeks' funnel plots; E: Fagan's nomogram. Figure S6: diagnostic performance of LSM for fibrosis grade ≥*F*2 in alcoholic liver disease/nonalcoholic fatty liver disease. A: positive likelihood; B: negative likelihood; C: diagnostic odds ratio; D: Deeks' funnel plots; E: Fagan's nomogram. Figure S7: diagnostic performance of LSM for fibrosis grade ≥*F*3 in alcoholic liver disease/nonalcoholic fatty liver disease. A: positive likelihood; B: negative likelihood; C: diagnostic odds ratio; D: Deeks' funnel plots; E: Fagan's nomogram. Figure S8: diagnostic performance of LSM for fibrosis grade =*F*4 in alcoholic liver disease/nonalcoholic fatty liver disease. A: positive likelihood; B: negative likelihood; C: diagnostic odds ratio; D: Deeks' funnel plots; E: Fagan's nomogram.
