# Supplementary material for: Transient Elastography in Alcoholic Liver Disease and Nonalcoholic Fatty Liver Disease: A Systemic Review and Meta-Analysis
Source: Can J Gastroenterol Hepatol. 2021 Jan 20;2021:8859338. doi: 10.1155/2021/8859338 (PMC7840258; doi:10.1155/2021/8859338)
Supplement: Supplementary Materials — Table S1: characteristics of the included studies. Table S2: the meta-regression analysis of CAP detecting patients with steatosis grade ≥S1. Table S3: the meta-regression analysis of CAP detecting patients with steatosis grade ≥S2. Table S4: the meta-regression analysis of CAP detecting patients with steatosis grade =S3. Table S5: the meta-regression analysis of CAP detecting patients with fibrosis grade ≥F1. Table S6: the meta-regression analysis of CAP detecting patients with fibrosis grade ≥F2. Table S7: the meta-regression analysis of CAP detecting patients with fibrosis grade ≥F3. Table S8: the meta-regression analysis of CAP detecting patients with fibrosis grade =F4. Figure S1: overall methodology quality assessment of included articles using the QUADAS-2 criteria. Figure S2: diagnostic performance of CAP for steatosis grade ≥S1 in alcoholic liver disease/nonalcoholic fatty liver disease. A: positive likelihood; B: negative likelihood; C: diagnostic odds ratio; D: Deeks' funnel plots; E: Fagan's nomogram. Figure S3: diagnostic performance of CAP for steatosis grade ≥S2 in alcoholic liver disease/nonalcoholic fatty liver disease. A: positive likelihood; B: negative likelihood; C: diagnostic odds ratio; D: Deeks' funnel plots; E: Fagan's nomogram. Figure S4: diagnostic performance of CAP for steatosis grade =S3 in alcoholic liver disease/nonalcoholic fatty liver disease. A: positive likelihood; B: negative likelihood; C: diagnostic odds ratio; D: Deeks' funnel plots; E: Fagan's nomogram. Figure S5: diagnostic performance of LSM for fibrosis grade ≥F1 in alcoholic liver disease/nonalcoholic fatty liver disease. A: positive likelihood; B: negative likelihood; C: diagnostic odds ratio; D: Deeks' funnel plots; E: Fagan's nomogram. Figure S6: diagnostic performance of LSM for fibrosis grade ≥F2 in alcoholic liver disease/nonalcoholic fatty liver disease. A: positive likelihood; B: negative likelihood; C: diagnostic odds ratio; D: Deeks' funnel plots; E: Fagan's nom [file 8859338.f1.docx]

**SUPPLEMENTARY MATERIALS**

**Retrieval strategies:**

**Pubmed:** ((((((Non alcoholic Fatty Liver Disease[Title/Abstract] OR NAFLD[Title/Abstract] OR Nonalcoholic Fatty Liver Disease[Title/Abstract] OR Fatty Liver, Nonalcoholic[Title/Abstract] OR Fatty Livers, Nonalcoholic[Title/Abstract] OR Liver, Nonalcoholic Fatty[Title/Abstract] OR Livers, Nonalcoholic Fatty[Title/Abstract] OR Nonalcoholic Fatty Liver；Nonalcoholic Fatty Livers[Title/Abstract] OR Nonalcoholic Steatohepatitis[Title/Abstract] OR Nonalcoholic Steatohepatitides[Title/Abstract] OR Steatohepatitides, Nonalcoholic[Title/Abstract] OR Steatohepatitis, Nonalcoholic[Title/Abstract] OR Non-alcoholic steatohapatitis[Title/Abstract] OR non alcoholic steatohapatitis[Title/Abstract] OR NASH[Title/Abstract] OR Non-alcoholic Fatty Liver Disease[Title/Abstract])) OR Non-alcoholic Fatty Liver Disease[MeSH Terms]）)) OR ((("Liver Diseases, Alcoholic"[Mesh]) OR (Alcoholic Liver Diseases[Title/Abstract] OR Alcoholic Liver Disease[Title/Abstract] OR Liver Disease, Alcoholic[Title/Abstract])) OR Alcoholic hepatitis[Title/Abstract]) OR ASH[Title/Abstract]) OR ALD[Title/Abstract]))))) AND (((transient elastography[Title/Abstract]) OR Controlled Attenuation Parameter[Title/Abstract]) OR liver stiffness[Title/Abstract])

**Embase:** ('nonalcoholic fatty liver'/exp OR 'alcohol liver disease'/exp OR 'non alcoholic fatty liver disease':ab,ti OR nafld:ab,ti OR 'nonalcoholic fatty liver disease':ab,ti OR 'fatty liver, nonalcoholic':ab,ti OR 'fatty livers, nonalcoholic':ab,ti OR 'liver, nonalcoholic fatty':ab,ti OR 'livers, nonalcoholic fatty':ab,ti OR 'nonalcoholic fatty liver':ab,ti OR 'nonalcoholic fatty livers':ab,ti OR 'nonalcoholic steatohepatitis':ab,ti OR 'nonalcoholic steatohepatitides':ab,ti OR 'steatohepatitides, nonalcoholic':ab,ti OR 'steatohepatitis, nonalcoholic':ab,ti OR 'non-alcoholic steatohapatitis':ab,ti OR 'non alcoholic steatohapatitis':ab,ti OR nash:ab,ti OR 'non-alcoholic fatty liver disease':ab,ti OR 'liver diseases, alcoholic':ab,ti OR 'alcoholic liver diseases':ab,ti OR 'liver disease, alcoholic':ab,ti OR 'alcoholic hepatitis':ab,ti OR ash:ab,ti OR ald:ab,ti) AND ('transient elastography'/exp OR 'controlled attenuation parameter'/exp OR 'liver stiffness'/exp OR 'transient elastography':ab,ti OR 'liver stiffness':ab,ti OR 'controlled attenuation parameter':ab,ti)

**Web of Science:** TOPIC: (Non alcoholic Fatty Liver Disease OR NAFLD OR Nonalcoholic Fatty Liver Disease OR Fatty Liver, Nonalcoholic OR Fatty Livers, Nonalcoholic OR Liver, Nonalcoholic Fatty OR Livers, Nonalcoholic Fatty OR Nonalcoholic Fatty Liver OR Nonalcoholic Fatty Livers OR Nonalcoholic Steatohepatitis OR Nonalcoholic Steatohepatitides OR Steatohepatitides, Nonalcoholic OR Steatohepatitis, Nonalcoholic OR Non-alcoholic steatohapatitis OR non alcoholic steatohapatitis OR NASH OR Non-alcoholic Fatty Liver Disease OR Liver Diseases, Alcoholic OR Alcoholic Liver Diseases OR Liver Disease, Alcoholic OR Alcoholic hepatitis OR ASH OR ALD) AND TOPIC: (transient elastography OR Controlled Attenuation Parameter OR liver stiffness)

**Cochrane:** ((MeSH descriptor: [Non-alcoholic Fatty Liver Disease] explode all trees) OR (Non alcoholic Fatty Liver Disease OR NAFLD OR Nonalcoholic Fatty Liver Disease OR Fatty Liver, Nonalcoholic OR Fatty Livers, Nonalcoholic OR Liver, Nonalcoholic Fatty OR Livers, Nonalcoholic Fatty OR Nonalcoholic Fatty Liver OR Nonalcoholic Fatty Livers OR Nonalcoholic Steatohepatitis OR Nonalcoholic Steatohepatitides OR Steatohepatitides, Nonalcoholic OR Steatohepatitis, Nonalcoholic OR Non-alcoholic steatohapatitis OR non alcoholic steatohapatitis OR NASH OR Non-alcoholic Fatty Liver Disease):ti,ab,kw) ) AND ((transient elastography):ti,ab,kw OR (Controlled Attenuation Parameter):ti,ab,kw OR (liver stiffness):ti,ab,kw)

**Table S1: characteristics of the included studies.**

| Study | Year | Country | Study design | Disease | Male (Number; %) | Age (years) | BMI (kg/m^2^) | Steatosis (CAP); Cut-off value (kPa) | Probe type (M/XL/Mix/Unknown) | SEN | SPE | Fibrosis (LSM); Cut-off value (kPa) | Probe type (M/XL/Mix/Unknown) | SEN | SPE |
| --- | --- | --- | --- | --- | --- | --- | --- | --- | --- | --- | --- | --- | --- | --- | --- |
| Wong et al [1] | 2019 | China/France | Prospective | NAFLD | 212 (43) | 54 ± 12 | 30.4 | / | / | / | / | F≥3 15.0  F≥4 15.0  F≥3 15.0  F=4 15.0 | M  M  XL  XL | 0.34  0.54  0.27  0.47 | 0.96  0.93  0.98  0.95 |
| Siddiqui et al [2] | 2019 | America | Prospective | NAFLD | 127 (32) | 51 ± 11 | 34.4 | S≥1 285  S≥2 311  S=3 306 | Mix  Mix  Mix | 0.80  0.77  0.80 | 0.77  0.57  0.40 | F≥1 8.6  F≥2 8.6  F≥3 8.6  F=4 13.1 | Mix  Mix  Mix  Mix | 0.53  0.66  0.70  0.89 | 0.87  0.80  0.74  0.86 |
| Eddowes et al [3] | 2019 | England | Prospective | NAFLD | 212 (55) | 54 ± 18 | 33.8 | S≥1 302  S≥2 331  S=3 337 | Mix  Mix  Mix | 0.80  0.70  0.72 | 0.83  0.76  0.63 | F≥2 8.2  F≥3 9.7  F=4 13.6 | Mix  Mix  Mix | 0.71  0.71  0.85 | 0.70  0.75  0.79 |
| Runge et al [4] | 2018 | Netherlands | Prospective | NAFLD | 40 (73) | 52.9 | 27.8 | S≥1 260  S≥2 296  S=3 334 | M  M  M | 0.90  0.93  0.78 | 0.60  0.56  0.76 | / | / | / | / |
| Ooi et al [5] | 2018 | Australia | Prospective | NAFLD | 45 (25) | 44 ± 12 | 45 | S≥2 285 | XL | 0.85 | 0.47 | F≥3 9 | XL | 1.00 | 0.47 |
| Garg et al [6] | 2018 | India | Prospective | NAFLD | 29 (23) | 39 ± 11 | 45.2 | S≥1 323  S≥2 336  S=3 357 | XL  XL  XL | 0.59  0.74  1.00 | 0.83  0.76  0.78 | F≥1 6  F≥2 7.25  F≥3 12.45 | XL  XL  XL | 0.80  0.70  0.64 | 0.56  0.59  0.88 |
| Voican et al [7] | 2017 | France | Prospective | ALD | 154 (80) | 48 ± 0.7 | 23 | / | / | / | / | F≥3 12  F=4 15 | M  M | 0.92  0.85 | 0.76  0.93 |
| Seki et al [8] | 2017 | Japan | Retrospective | NAFLD | 38 (61) | 51 ± 14 | 27.7 | / | / | / | / | F≥3 10 | M | 0.90 | 0.88 |
| Petta et al [9] | 2017 | Italy | Prospective | NAFLD | 144 (44) | 54 ± 13 | / | / | / | / | / | F≥2 8.5  F≥3 10.1 | Unknown  Unknown | 0.74  0.78 | 0.74  0.78 |
| Petta et al [10] | 2017 | Italy | Prospective | NAFLD | 458 (60) | 51 ± 13 | 29.6 | / | / | / | / | F≥3 9.6  F≥3 9.6  F≥3 9.6 | M  M  M | 0.74  0.72  0.75 | 0.81  0.86  0.78 |
| Loong et al [11] | 2017 | China | Prospective | NAFLD | 119 (55) | 52 | 26.8 | / | / | / | / | F≥2 9.0  F≥3 9.6 | M  M | 0.65  0.84 | 0.88  0.87 |
| Forlano et al [12] | 2017 | England | Prospective | NAFLD | / | / | / | S≥1 250 | / | 0.90 | 0.60 | / | / | / | / |
| Chan et al [13] | 2017 | Malaysia | Prospective | NAFLD | 11 (50) | 20 ± 1.3 | 27.5 | S≥1 266  S≥2 266  S=3 267  S≥1 271  S≥2 271  S=3 304 | M  M  M  XL  XL  XL | 0.91  0.91  1.00  0.95  0.95  0.80 | 0.87  0.87  0.41  0.91  0.61  0.55 | F≥1 7.1  F≥2 10.7  F≥3 13.6  F=4 15.1  F≥1 5.9  F≥2 8.9  F≥3 11.5  F=4 12.4 | M  M  M  M  XL  XL  XL  XL | 0.79  0.85  0.88  1.00  0.85  0.44  0.88  1.00 | 0.80  0.89  0.97  0.96  0.76  0.93  0.97  0.95 |
| Thiele et al [14] | 2016 | Danish | Prospective | ALD | 145 (74) | 55 ± 11 | / | / | / | / | / | F≥2 9.6  F=4 19.7 | Unknown  Unknown | 0.83  0.97 | 0.91  0.90 |
| Tapper et al [15] | 2016 | America | Prospective | NAFLD | 96 (59) | 51 ± 13 | 32.2 | / | / | / | / | F≥3 9.9 | M | 0.95 | 0.77 |
| Mota et al [16] | 2016 | America | Prospective | NASH | 18 (32) | 52.8 | / | / | / | / | / | F=4 11.8 | Mix | 0.83 | 0.66 |
| Lee et al [17] | 2016 | Korea | Prospective | NAFLD | 111 (61) | 41 ± 14 | 27.9 | S≥1 247  S≥2 280  S=3 300 | Mix  Mix  Mix | 0.88  0.85  0.73 | 1.00  0.80  0.61 | F≥1 6.7  F≥2 8.0  F≥3 9.0  F=4 11.0 | Mix  Mix  Mix  Mix | 0.66  0.83  0.96  1.00 | 0.85  0.85  0.86  0.90 |
| Imajo et al [18] | 2016 | Japan | Retrospective | NAFLD | 87 (57) | 57.1 | 28.1 | S≥1 236  S≥2 270  S=3 302 | M  M  M | 0.82  0.78  0.64 | 0.91  0.81  0.74 | F≥1 7.0  F≥2 11.0  F≥3 11.4  F=4 14.0 | M  M  M  M | 0.62  0.65  0.86  1.00 | 1.00  0.89  0.84  0.76 |
| Cassinotto et al [19] | 2016 | France | Prospective | NAFLD | 172 (59) | 57 ± 12 | >30 | / | / | / | / | F≥2 6.2  F≥3 8.2  F=4 9.5 | M  M  M | 0.90  0.90  0.92 | 0.45  0.61  0.62 |
| Boursier et al [20] | 2016 | France | Prospective | NAFLD | 337 (47) | 56 ± 12 | 31.1 | / | / | / | / | F≥3 8.7 | M | 0.88 | 0.63 |
| Wong et al [21] | 2016 | China | Prospective | NAFLD | 158 (54) | 50 ± 10 | 27.7 | / | / | / | / | F≥3 9.6 | / | 0.72 | 0.86 |
| Petta et al [22] | 2015 | Italy | Retrospective | NAFLD | 121 (68)  102 (72) | 45 ± 13  44 ± 12 | 29.3  27.4 | / | / | / | / | F≥3 9.6  F≥3 9.6 | M  M | 0.85  0.68 | 1.00  0.86 |
| Pathik et al [23] | 2015 | India | Prospective | NAFLD | 196 (66) | 41.9 | 29.15 | / | / | / | / | F≥3 12.0 | M | 0.90 | 0.80 |
| Hiriart et al [24] | 2015 | France | Prospective | NAFLD | 154 (59) | 56 | 30.2 | S≥2 310 | M | 0.79 | 0.71 | / | / | / | / |
| Fernandez et al [25] | 2015 | Belgium | Retrospective | ALD | 94 (70) | 46 ± 0.9 | 26.1 | / | / | / | / | F≥3 10.3  F=4 18.0 | Unknown  Unknown | 0.91  0.90 | 0.67  0.86 |
| Dincses et al [26] | 2015 | Turkey | Retrospective | NAFLD | 30 (58) | 45 ± 9 | 30.8 | / | / | / | / | F≥2 /  F≥3 / | Unknown  Unknown | 0.75  1.00 | 0.78  0.76 |
| Vuppalanchi et al [27] | 2014 | America | Prospective | NAFLD | 38 (40) | 54 | 31 | / | / | / | / | F≥2 10.3 | Unknown | 0.80 | 0.75 |
| Naveau et al [28] | 2014 | France | Prospective | NAFLD | 19 (19) | 42 ± 0.5 | 42.3 | / | / | / | / | F≥2 7.6  F≥3 7.6 | M/XL  M/XL | 0.76  1.00 | 0.78  0.74 |
| Karlas et al [29] | 2014 | Germany | Prospective | NAFLD | 31 (48) | 51.4 | 27.5 | S≥1 233.5  S≥2 268.5  S=3 301.2 | M  M  M | 0.93  0.97  0.82 | 0.87  0.81  0.76 | F≥2 8.85 | M | 1.00 | 0.98 |
| Chan et al [30] | 2014 | Malaysia | Prospective | NAFLD | 74 (46) | 40.5 | 26.2 | S≥1 263  S=2 263  S=3 281 | M  M  M | 0.92  0.97  1.00 | 0.94  0.68  0.53 | / | / | / | / |
| Aykut et al [31] | 2014 | Turkey | Prospective | NAFLD | 50 (57) | 46 ± 9 | 30.3 | / | / | / | / | F≥2 /  F≥3 /  F=4 / | Mix  Mix  Mix | 0.75  0.96  1.00 | 0.93  0.90  0.76 |
| Mahadeva et al [32] | 2013 | Malaysia/China/India | Prospective | NAFLD | 69 (53) | 50 ± 12 | / | / | / | / | / | F≥2 6.85  F≥3 7.1  F=4 11.3 | M  M  M | 0.59  0.70  0.88 | 0.69  0.67  0.89 |
| Kumar et al [33] | 2013 | India | Prospective | NAFLD | 90 (75) | 39 ± 13 | 26.1 | / | / | / | / | F≥1 6.1  F≥2 7.0  F≥3 9.0  F=4 11.8 | M  M  M  M | 0.78  0.77  0.85  0.90 | 0.68  0.78  0.88  0.88 |
| Alkhouri et al [34] | 2013 | Italy | Prospective | NAFLD | 46 (69) | 8.5 | / | / | / | / | / | F≥2 8.6 | S | 1.00 | 1.00 |
| Wong et al [35] | 2012 | France/China | Prospective | NAFLD | 110 (57) | 52 ± 11 | 28.9 | / | / | / | / | F≥2 7.0  F≥3 8.7  F=4 10.3  F≥2 6.2  F≥3 7.2  F=4 7.9 | M  M  M  XL  XL  XL | 0.79  0.83  0.81  0.73  0.78  0.88 | 0.64  0.78  0.83  0.66  0.78  0.76 |
| Friedrich-Rust et al [36] | 2012 | Germany | Prospective | NAFLD | 30 (53) | 45 ± 14 | 28 | S≥2 245  S=3 301 | Mix  Mix | 0.97  0.76 | 0.67  0.68 | / | / | / | / |
| Petta et al [37] | 2011 | Italy | Prospective | NAFLD | 104 (71) | 44 ± 13 | 29.1 | / | / | / | / | F≥2 7.25  F≥3 8.75 | M  M | 0.69  0.76 | 0.71  0.78 |
| Gaia et al [38] | 2011 | Italy | Prospective | NAFLD | 52 (72) | 48 | 27.2 | / | / | / | / | F≥1 5.5  F≥2 7.0  F≥3 8.0  F=4 10.5 | Mix  Mix  Mix  Mix | 0.57  0.80  0.80  0.96 | 0.80  0.75  0.48  0.70 |
| Wong et al [39] | 2010 | France/China | Prospective | NAFLD | 135 (55) | 51 ± 11 | 28 | / | / | / | / | F≥2 7.0  F≥3 8.7  F=4 10.3 | Mix  Mix  Mix | 0.79  0.84  0.92 | 0.76  0.83  0.88 |
| Mueller et al [40] | 2010 | Germany | Prospective | ALD | 73 (72) | 53 ± 11 | 25.4 | / | / | / | / | F≥3 8.0  F=4 11.5 | M  M | 0.91  1.00 | 0.75  0.77 |
| Mahadeva et al [41] | 2010 | Malaysia | Prospective | NAFLD | 15 (60) | 49 ± 12 | 26.5 | / | / | / | / | F≥3 9.4 | Unknown | 0.83 | 0.89 |
| Lupsor et al [42] | 2010 | Roman | Prospective | NASH | 51 (71) | 42 ± 6 | 28.7 | / | / | / | / | F≥1 5.3  F≥2 6.8  F=3 10.4 | Unknown  Unknown  Unknown | 0.93  0.67  1.00 | 0.78  0.84  0.97 |
| De Ledinghen et al [43] | 2009 | France | Prospective | NAFLD | 115 (55) | 51 | 28 | / | / | / | / | F≥2 7.0  F≥3 8.7  F=4 10.3 | Mix  Mix  Mix | 0.77  0.84  0.95 | 0.77  0.87  0.88 |
| Yoneda et al [44] | 2008 | Japan | Prospective | NAFLD | 40 (41) | 52 ± 14 | 26.6 | / | / | / | / | F≥1 5.9  F≥2 6.7  F≥3 9.8  F=4 17.5 | Mix  Mix  Mix  Mix | 0.86  0.88  0.86  1.00 | 0.89  0.74  0.81  1.00 |
| Nobili et al [45] | 2008 | Italy | Prospective | NAFLD | 31 (62) | 13 | 25.7 | / | / | / | / | F≥1 5.1  F≥2 7.4  F≥3 10.2 | M  M  M | 0.97  1.00  1.00 | 0.91  0.92  1.00 |
| Nguyen-Khac et al [46] | 2008 | France | Prospective | ALD | 76 (74) | 53 ± 10 | 27.7 | / | / | / | / | F≥1 5.9  F≥2 7.8  F≥3 11.0  F=4 19.5 | Unknown  Unknown  Unknown  Unknown | 0.83  0.80  0.87  0.86 | 0.86  0.91  0.81  0.84 |
| Nahon et al [47] | 2008 | France | Prospective | ALD | 112 (76) | 54 ± 9 | 25.6 | / | / | / | / | F≥3 11.6  F=4 22.7 | Unknown  Unknown | 0.87  0.84 | 0.89  0.83 |
| Thiele et al [48] | 2018 | Denmark | Prospective | ALD | 149 (67) | 53 ± 11 | 26.0 | S≥1 270  S≥2 290  S=3 290 | Unknown  Unknown  Unknown | 0.70  0.64  0.88 | 0.73  0.74  0.67 | F≥3 13.2 | Unknown | 0.91 | 0.94 |
| Naveau et al [49] | 2017 | France | Retrospective  Prospective | NAFLD  NAFLD | 43 (22)  31 (26) | 41 ± 1  40 ± 1 | 44  44 | S≥1 308  S≥2 335  S=3 341  S≥1 298  S≥2 303  S=3 326 | Mix  Mix  Mix  Mix  Mix  Mix | 0.68  0.65  0.74  0.78  0.90  0.83 | 0.69  0.79  0.74  0.83  0.69  0.71 | / | / | / | / |
| Lee et al [50] | 2017 | Korea | Prospective | NAFLD | 41 (44) | 56 ± 3 | 27.1 | / | / | / | / | F≥2 7.4  F≥3 8.0  F=4 10.8 | M  M  M | 0.63  0.83  0.92 | 0.92  0.85  0.81 |
| Ergelen et al [51] | 2016 | Turkey | Prospective | NASH | 39 (62) | 47.1 | 30.4 | / | / | / | / | F≥2 9.8 | Mix | 0.90 | 0.91 |
| De Ledinghen et al [52] | 2016 | France | Prospective | NAFLD | 46 (55) | 57 ± 10 | 30.2 | S≥2 310  S=3 311 | M  M | 0.79  0.87 | 0.71  0.47 | / | / | / | / |
| Attia et al [53] | 2016 | Germany | Prospective  Prospective | NAFLD  NAFLD | 31 (51)  15 (58) | 51 ± 13  53 ± 10 | 28  36 | / | / | / | / | F≥2 7.0  F≥3 11.8  F=4 15.0  F≥2 6.7  F≥3 9.3  F=4 11.7 | Mix  Mix  Mix  Mix  Mix  Mix | 0.85  0.79  1.00  0.87  0.91  1.00 | 0.80  0.94  0.93  0.76  0.80  0.83 |
| Ergelen et al [54] | 2015 | Turkey | Prospective | NAFLD | 43 (49) | 46 ± 9 | 30.6 | / | / | / | / | F≥3 9.6  F=4 9.9 | Mix  Mix | 0.68  0.86 | 0.90  0.77 |
| Kumar et al [55] | 2013 | India | Prospective | NAFLD | 46 (73) | 37 ± 6 | 26 | S≥2 258  S=3 283 | Unknown  Unknown | 0.78  0.71 | 0.73  0.68 | / | / | / | / |
| Fernandez et al [56] | 2012 | Belgium | Retrospective | ALD | 95 (68) | 54 ± 0.9 | / | / | / | / | / | F≥3 10.5  F=4 15.7 | Unknown  Unknown | 0.91  0.90 | 0.67  0.87 |
| Yoneda et al [57] | 2010 | Japan | Prospective | NAFLD | 25 (46) | 50.6 | 27.1 | / | / | / | / | F≥3 9.9  F=4 16.0 | Unknown  Unknown | 1.00  1.00 | 0.93  0.98 |
| Myers et al [58] | 2010 | Canada | Retrospective | NAFLD | / | / | / | / | / | / | / | F≥2 7.7  F≥3 10.3  F=4 11.1 | Unknown  Unknown  Unknown | 0.94  0.70  1.00 | 0.61  0.76  0.82 |
| Janssens et al [59] | 2010 | Belgium | Prospective | ALD | 34 (69) | 53 | 25 | / | / | / | / | F≥3 17  F=4 19.6 | Unknown  Unknown | 0.72  0.80 | 0.77  0.76 |
| Kim et al [60] | 2009 | Korea | Prospective | ALD | 37 (82) | 46 | 23 | / | / | / | / | F≥3 9.5  F=4 12.5 | M  M | 0.97  1.00 | 0.78  0.50 |
| Lemoine et al [61] | 2008 | France | Retrospective | NAFLD | 40 (83) | 56 ± 13 | / | / | / | / | / | F=4 34.9 | Unknown | 0.90 | 0.88 |
| Chang et al [62] | 2018 | Singapore | Prospective | NASH | / | 49 ± 12 | 23.9 | / | / | / | / | F≥2 11  F=4 15 | Mix  Mix | 0.94  1.00 | 0.77  0.81 |

NAFLD: Non-alcoholic fatty liver disease; ALD: Alcoholic liver disease; NASH: Non-alcoholic steatohepatitis; BMI: Body mass index; CAP: Controlled attenuation parameter; LSM: Liver stiffness measurement; SEN: Sensitivity; SPE: Specificity.

| **Section/topic** | **#** | **Checklist item** | **Reported on page #** |
| --- | --- | --- | --- |
| **TITLE** | | |  |
| Title | 1 | Identify the report as a systematic review, meta-analysis, or both. | 1 |
| **ABSTRACT** | | |  |
| Structured summary | 2 | Provide a structured summary including, as applicable: background; objectives; data sources; study eligibility criteria, participants, and interventions; study appraisal and synthesis methods; results; limitations; conclusions and implications of key findings; systematic review registration number. | 2 |
| **INTRODUCTION** | | |  |
| Rationale | 3 | Describe the rationale for the review in the context of what is already known. | 3 |
| Objectives | 4 | Provide an explicit statement of questions being addressed with reference to participants, interventions, comparisons, outcomes, and study design (PICOS). | 3 |
| **METHODS** | | |  |
| Protocol and registration | 5 | Indicate if a review protocol exists, if and where it can be accessed (e.g., Web address), and, if available, provide registration information including registration number. | 4 |
| Eligibility criteria | 6 | Specify study characteristics (e.g., PICOS, length of follow-up) and report characteristics (e.g., years considered, language, publication status) used as criteria for eligibility, giving rationale. | 4 |
| Information sources | 7 | Describe all information sources (e.g., databases with dates of coverage, contact with study authors to identify additional studies) in the search and date last searched. | 4 |
| Search | 8 | Present full electronic search strategy for at least one database, including any limits used, such that it could be repeated. | 4 |
| Study selection | 9 | State the process for selecting studies (i.e., screening, eligibility, included in systematic review, and, if applicable, included in the meta-analysis). | 4 |
| Data collection process | 10 | Describe method of data extraction from reports (e.g., piloted forms, independently, in duplicate) and any processes for obtaining and confirming data from investigators. | 4-5 |
| Data items | 11 | List and define all variables for which data were sought (e.g., PICOS, funding sources) and any assumptions and simplifications made. | 4-5 |
| Risk of bias in individual studies | 12 | Describe methods used for assessing risk of bias of individual studies (including specification of whether this was done at the study or outcome level), and how this information is to be used in any data synthesis. | 4-5 |
| Summary measures | 13 | State the principal summary measures (e.g., risk ratio, difference in means). | 4-5 |
| Synthesis of results | 14 | Describe the methods of handling data and combining results of studies, if done, including measures of consistency (e.g., I^2^) for each meta-analysis. | 4-5 |

| **Section/topic** | **#** | **Checklist item** | **Reported on page #** |
| --- | --- | --- | --- |
| Risk of bias across studies | 15 | Specify any assessment of risk of bias that may affect the cumulative evidence (e.g., publication bias, selective reporting within studies). | 4-5 |
| Additional analyses | 16 | Describe methods of additional analyses (e.g., sensitivity or subgroup analyses, meta-regression), if done, indicating which were pre-specified. | 4-5 |
| **RESULTS** | | |  |
| Study selection | 17 | Give numbers of studies screened, assessed for eligibility, and included in the review, with reasons for exclusions at each stage, ideally with a flow diagram. | 6-7 |
| Study characteristics | 18 | For each study, present characteristics for which data were extracted (e.g., study size, PICOS, follow-up period) and provide the citations. | 6-7 |
| Risk of bias within studies | 19 | Present data on risk of bias of each study and, if available, any outcome level assessment (see item 12). | 6-7 |
| Results of individual studies | 20 | For all outcomes considered (benefits or harms), present, for each study: (a) simple summary data for each intervention group (b) effect estimates and confidence intervals, ideally with a forest plot. | 6-7 |
| Synthesis of results | 21 | Present results of each meta-analysis done, including confidence intervals and measures of consistency. | 6-7 |
| Risk of bias across studies | 22 | Present results of any assessment of risk of bias across studies (see Item 15). | 6-7 |
| Additional analysis | 23 | Give results of additional analyses, if done (e.g., sensitivity or subgroup analyses, meta-regression [see Item 16]). | 6-7 |
| **DISCUSSION** | | |  |
| Summary of evidence | 24 | Summarize the main findings including the strength of evidence for each main outcome; consider their relevance to key groups (e.g., healthcare providers, users, and policy makers). | 8-9 |
| Limitations | 25 | Discuss limitations at study and outcome level (e.g., risk of bias), and at review-level (e.g., incomplete retrieval of identified research, reporting bias). | 9 |
| Conclusions | 26 | Provide a general interpretation of the results in the context of other evidence, and implications for future research. | 9 |
| **FUNDING** | | |  |
| Funding | 27 | Describe sources of funding for the systematic review and other support (e.g., supply of data); role of funders for the systematic review. | 10 |

**Table S2:** The meta-regression analysis of CAP detecting patients with **steatosis grade ≥ S1.**

LogOR was used as response variables as well as BMI, cut-off value, sample size, ethnicity, disease, ethnicity, and study design were as covariates. Estimate of between-study variance tau2 = 0. Residual variation due to heterogeneity: I-squared_res = 0%. Proportion of between-study variance explained: Adj R-squared = 100.00%. Joint test for all covariates with Knapp-Hartung modifcation: Prob > F = 0. 0144.

| LogOR | Coef. | Std. Err. | t | P>\|t\| | [95% Conf. Interval] | |
| --- | --- | --- | --- | --- | --- | --- |
| BMI | -1.57799 | 0.9813595 | -1.61 | 0.159 | -3.979291 | 0.8233099 |
| Cut-off value | -0.0678535 | 0.7986199 | -0.08 | 0.935 | -2.022006 | 1.886299 |
| Sample size | 0.8929285 | 0.6535977 | 1.37 | 0.221 | -0.7063674 | 2.492224 |
| Ethnicity | 0.9545966 | 0.5932803 | 1.61 | 0.159 | -0.497108 | 2.406301 |
| Disease | 2.561581 | 1.017258 | 2.52 | 0.045 | 0.0724415 | 5.050721 |
| Design | 1.091018 | 0.5053059 | 2.16 | 0.074 | -0.1454213 | 2.327457 |

**Table S3:** The meta-regression analysis of CAP detecting patients with **steatosis grade ≥ S2.**

| LogOR | Coef. | Std. Err. | t | P>\|t\| | [95% Conf. Interval] | |
| --- | --- | --- | --- | --- | --- | --- |
| BMI | -0.6191239 | 0.653438 | -0.95 | 0.366 | -2.075075 | 0.8368267 |
| Cut-off value | -0.4074031 | 0.5218007 | -0.78 | 0.453 | -1.570047 | 0.7552413 |
| Sample size | -0.5378147 | 0.5595822 | -0.96 | 0.359 | -1.784642 | 0.7090122 |
| Ethnicity | -0.1477588 | 0.5802653 | -0.25 | 0.804 | -1.44067 | 1.145153 |
| Disease | 0.9675687 | 0.9137601 | 1.06 | 0.315 | -1.068416 | 3.003553 |
| Design | -0.1899811 | 0.5700357 | -0.33 | 0.746 | -1.4601 | 1.080137 |

LogOR was used as response variables as well as BMI, cut-off value, sample size, ethnicity, disease, ethnicity, and study design were as covariates. Estimate of between-study variance tau2 = 0.2528. Residual variation due to heterogeneity: I-squared_res = 57.43%. Proportion of between-study variance explained: Adj R-squared = 23.84%. Joint test for all covariates with Knapp-Hartung modifcation: Prob > F = 0. 2422.

**Table S4:** The meta-regression analysis of CAP detecting patients with **steatosis grade = S3.**

| LogOR | Coef. | Std. Err. | t | P>\|t\| | [95% Conf. Interval] | |
| --- | --- | --- | --- | --- | --- | --- |
| BMI | -0.5055553 | 0.5329353 | -0.95 | 0.371 | -1.734506 | 0.7233957 |
| Cut-off value | 0.2836796 | 0.6124243 | 0.46 | 0.656 | -1.128573 | 1.695933 |
| Sample size | -0.6552351 | 0.4968137 | -1.32 | 0.224 | -1.80089 | 0.4904193 |
| Ethnicity | -0.1472473 | 0.6509033 | -0.23 | 0.827 | -1.648233 | 1.353738 |
| Disease | -1.117353 | 0.9659542 | -1.16 | 0.281 | -3.344848 | 1.110141 |
| Design | -0.6497872 | 0.3698215 | -1.76 | 0.117 | -1.502597 | 0.2030227 |

LogOR was used as response variables as well as BMI, cut-off value, sample size, ethnicity, disease, ethnicity, and study design were as covariates. Estimate of between-study variance tau2 = 0.01878. Residual variation due to heterogeneity: I-squared_res = 0.0%. Proportion of between-study variance explained: Adj R-squared = 82.02%. Joint test for all covariates with Knapp-Hartung modifcation: Prob > F = 0. 2660.

| LogOR | Coef. | Std. Err. | t | P>\|t\| | [95% Conf. Interval] | |
| --- | --- | --- | --- | --- | --- | --- |
| BMI | -.9642858 | 1.018113 | -0.95 | 0.397 | -3.791021 | 1.862449 |
| Cut-off value | 0.234821 | 0.9827985 | 0.24 | 0.823 | -2.493865 | 2.963507 |
| Sample size | -0.5137695 | 0.9684833 | -0.53 | 0.624 | -3.20271 | 2.175171 |
| Ethnicity | -0.5958808 | 0.8870046 | -0.67 | 0.539 | -3.0586 | 1.866839 |
| Disease | -0.8137877 | 1.989967 | -0.41 | 0.704 | -6.338822 | 4.711247 |
| Design | -2.437444 | 2.206545 | -1.10 | 0.331 | -8.563795 | 3.688906 |

**Table S5:** The meta-regression analysis of CAP detecting patients with **fibrosis grade ≥ F1.**

LogOR was used as response variables as well as BMI, cut-off value, sample size, ethnicity, disease, ethnicity, and study design were as covariates. Estimate of between-study variance tau2 = 0.909. Residual variation due to heterogeneity: I-squared_res = 65.17%. Proportion of between-study variance explained: Adj R-squared = -745.09%. Joint test for all covariates with Knapp-Hartung modifcation: Prob > F = 0.8478.

**Table S6:** The meta-regression analysis of CAP detecting patients with **fibrosis grade ≥ F2.**

| LogOR | Coef. | Std. Err. | t | P>\|t\| | [95% Conf. Interval] | |
| --- | --- | --- | --- | --- | --- | --- |
| Sample size | -0.269343 | 0.2756374 | -0.98 | 0.335 | -0.8289167 | 0.2902307 |
| Ethnicity | 0.0855996 | 0.2975607 | 0.29 | 0.775 | -0.5184806 | 0.6896799 |
| Disease | -1.333057 | 0.6654851 | -2.00 | 0.053 | -2.684064 | 0.0179495 |
| Design | -0.2829257 | 0.4608666 | -0.61 | 0.543 | -1.218535 | 0.6526832 |

LogOR was used as response variables as well as sample size, ethnicity, disease, and study design were as covariates. Estimate of between-study variance tau2 = 0.2558. Residual variation due to heterogeneity: I-squared_res = 13.81%. Proportion of between-study variance explained: Adj R-squared = -745.09%. Joint test for all covariates with Knapp-Hartung modifcation: Prob > F = 0.3376.

**Table S7:** The meta-regression analysis of CAP detecting patients with **fibrosis grade ≥ F3.**

| LogOR | Coef. | Std. Err. | t | P>\|t\| | [95% Conf. Interval] | |
| --- | --- | --- | --- | --- | --- | --- |
| Sample size | -0.461945 | 0.3108041 | -1.49 | 0.144 | -1.087561 | 0.1636712 |
| Ethnicity | 0.4899646 | 0.2665769 | 1.84 | 0.073 | -0.0466269 | 1.026556 |
| Disease | 0.6779654 | 0.3238253 | 2.09 | 0.042 | 0.0261389 | 1.329792 |
| Design | 0.0729291 | 0.3540737 | 0.21 | 0.838 | -0.6397843 | 0.7856425 |

LogOR was used as response variables as well as sample size, ethnicity, disease, and study design were as covariates. Estimate of between-study variance tau2 = 0. 2118. Residual variation due to heterogeneity: I-squared_res = 51.77%. Proportion of between-study variance explained: Adj R-squared = 33.66%. Joint test for all covariates with Knapp-Hartung modifcation: Prob > F = 0.0899.

**Table S8:** The meta-regression analysis of CAP detecting patients with **fibrosis grade = F4.**

| LogOR | Coef. | Std. Err. | t | P>\|t\| | [95% Conf. Interval] | |
| --- | --- | --- | --- | --- | --- | --- |
| Sample size | -0.1792556 | 0.4243553 | -0.42 | 0.676 | -1.04716 | 0.6886484 |
| Ethnicity | 0.1086493 | 0.4232946 | 0.26 | 0.799 | -0.7570853 | 0.9743839 |
| Disease | 0.1331024 | 0.4389031 | 0.30 | 0.764 | -0.7645551 | 1.03076 |
| Design | 0.3520408 | 0.5458948 | 0.64 | 0.524 | -0.7644393 | 1.468521 |

LogOR was used as response variables as well as sample size, ethnicity, disease, and study design were as covariates. Estimate of between-study variance tau2 = 0. 3463. Residual variation due to heterogeneity: I-squared_res = 35.36%. Proportion of between-study variance explained: Adj R-squared = -27.16%. Joint test for all covariates with Knapp-Hartung modifcation: Prob > F = 0. 9385.


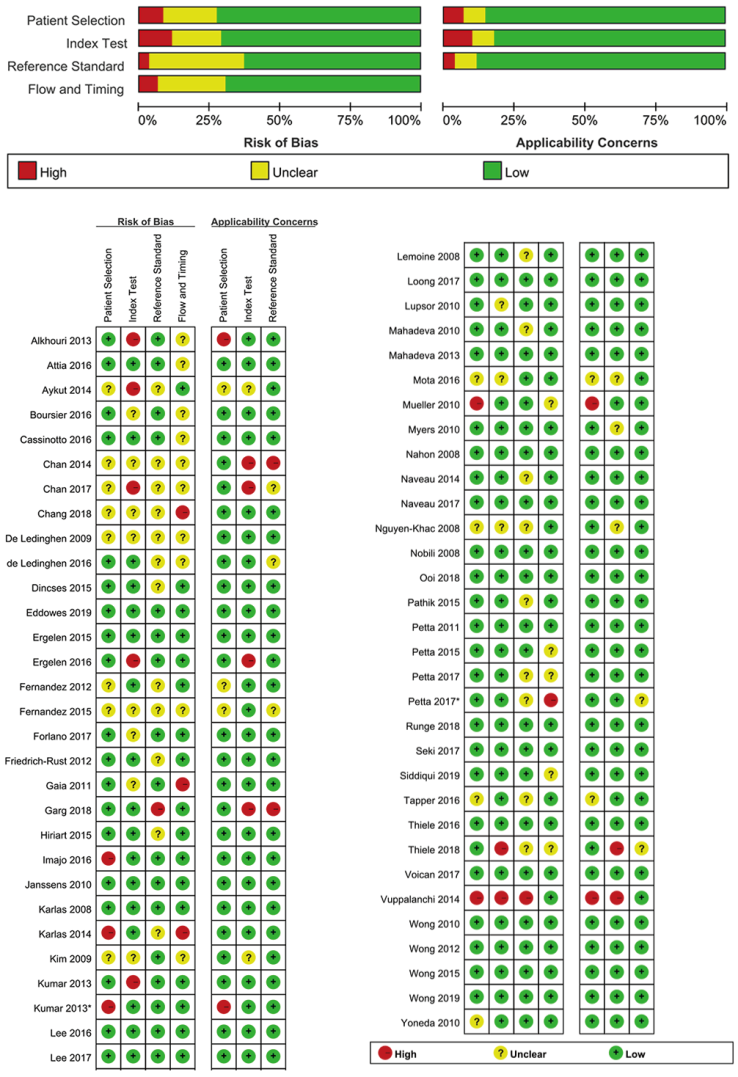


**Figure S1** Overall methodology quality assessment of included articles using the QUADAS-2 criteria.**
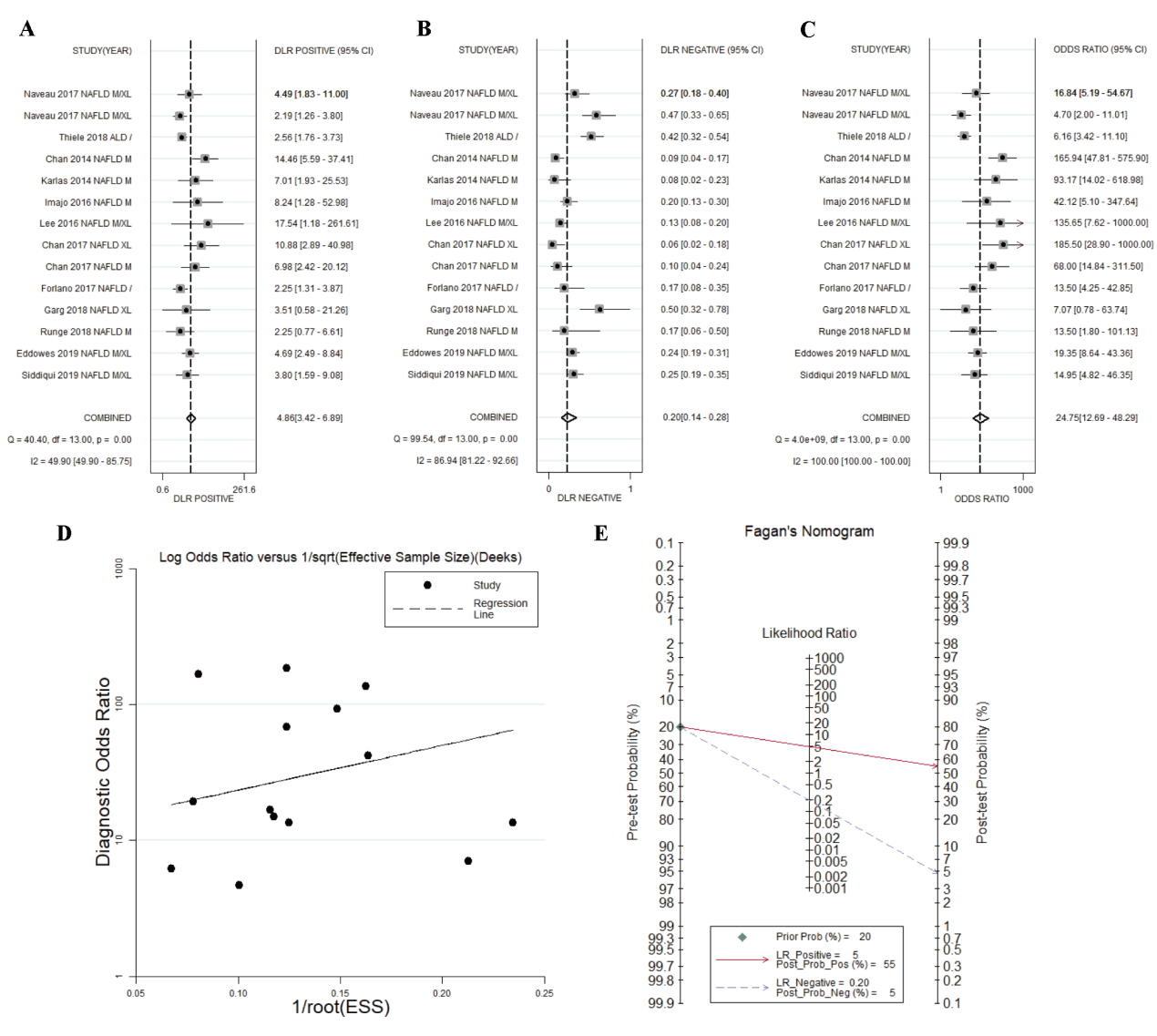
**

**Figure S2** Diagnostic performance of CAP for steatosis grade ≥ S1 in alcoholic liver disease/non-alcoholic fatty liver disease. A: Positive likelihood; B: Negative likelihood; C: Diagnostic odds ratio; D: Deeks’ funnel plots; E: Fagan’s Nomogram.

**
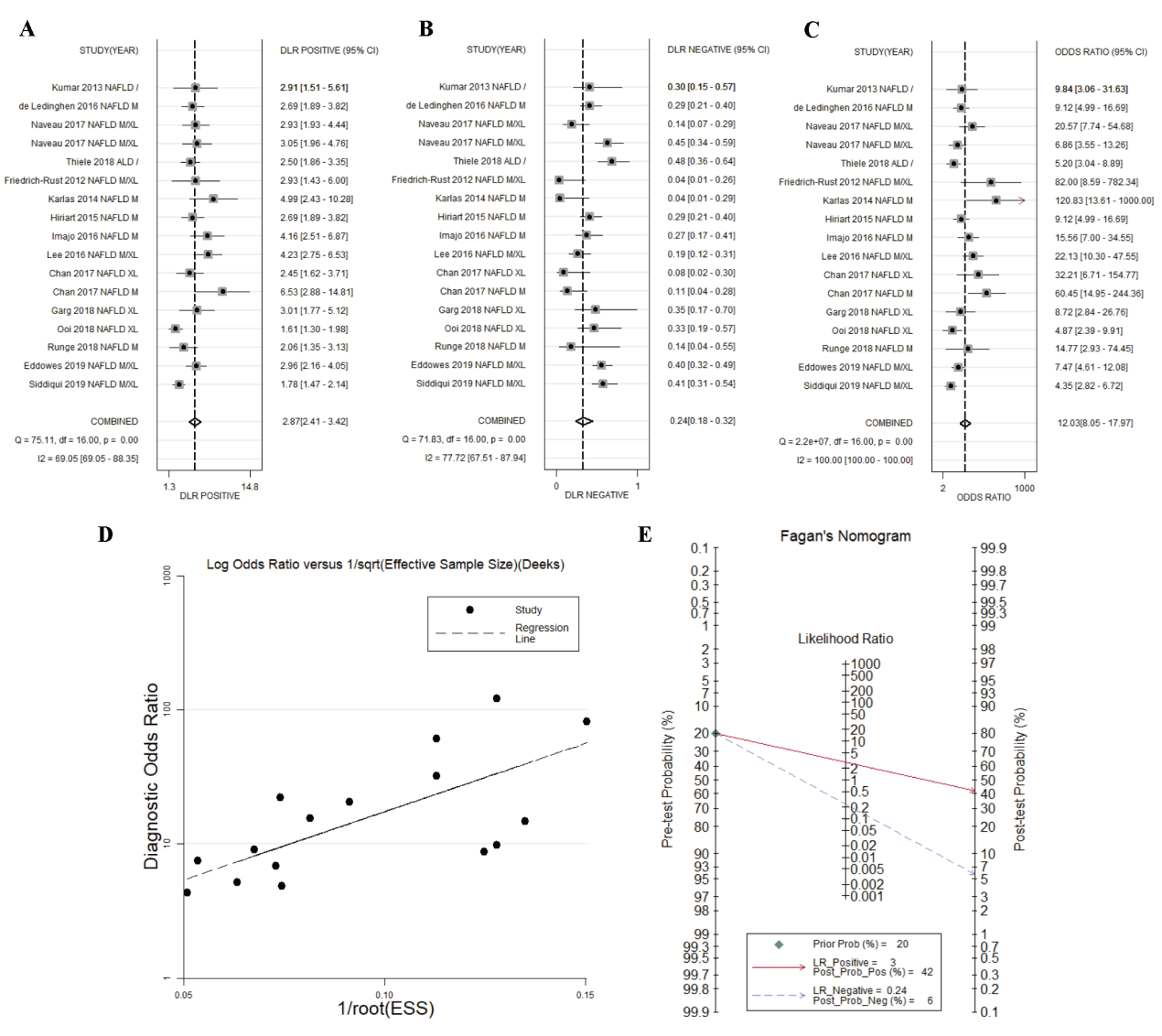
**

**Figure S3** Diagnostic performance of CAP for steatosis grade ≥ S2 in alcoholic liver disease/non-alcoholic fatty liver disease. A: Positive likelihood; B: Negative likelihood; C: Diagnostic odds ratio; D: Deeks’ funnel plots; E: Fagan’s Nomogram.

**
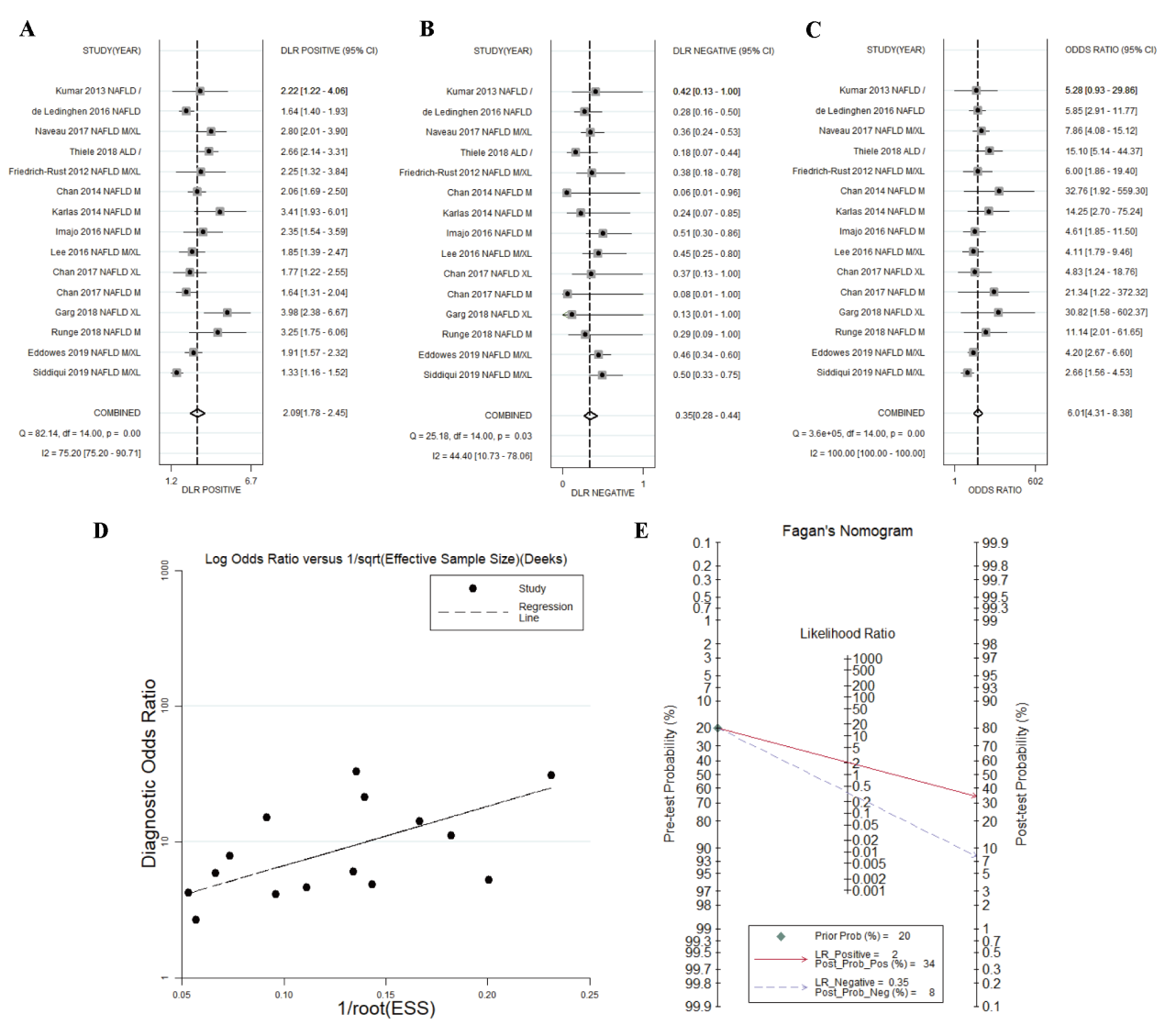
**

**Figure S4** Diagnostic performance of CAP for steatosis grade = S3 in alcoholic liver disease/non-alcoholic fatty liver disease. A: Positive likelihood; B: Negative likelihood; C: Diagnostic odds ratio; D: Deeks’ funnel plots; E: Fagan’s Nomogram.

**
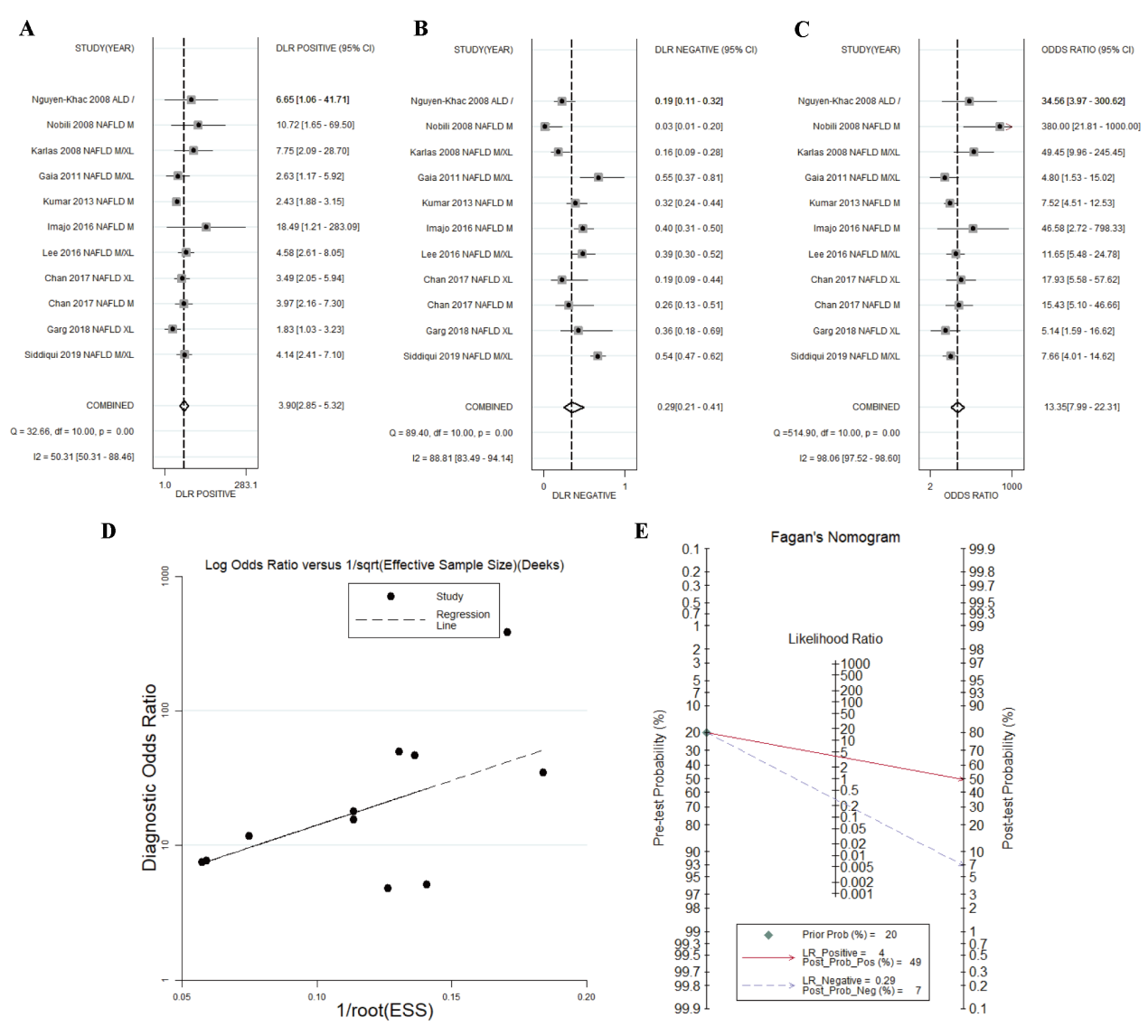
**

**Figure S5** Diagnostic performance of LSM for fibrosis grade ≥ F1 in alcoholic liver disease/non-alcoholic fatty liver disease. A: Positive likelihood; B: Negative likelihood; C: Diagnostic odds ratio; D: Deeks’ funnel plots; E: Fagan’s Nomogram.

**
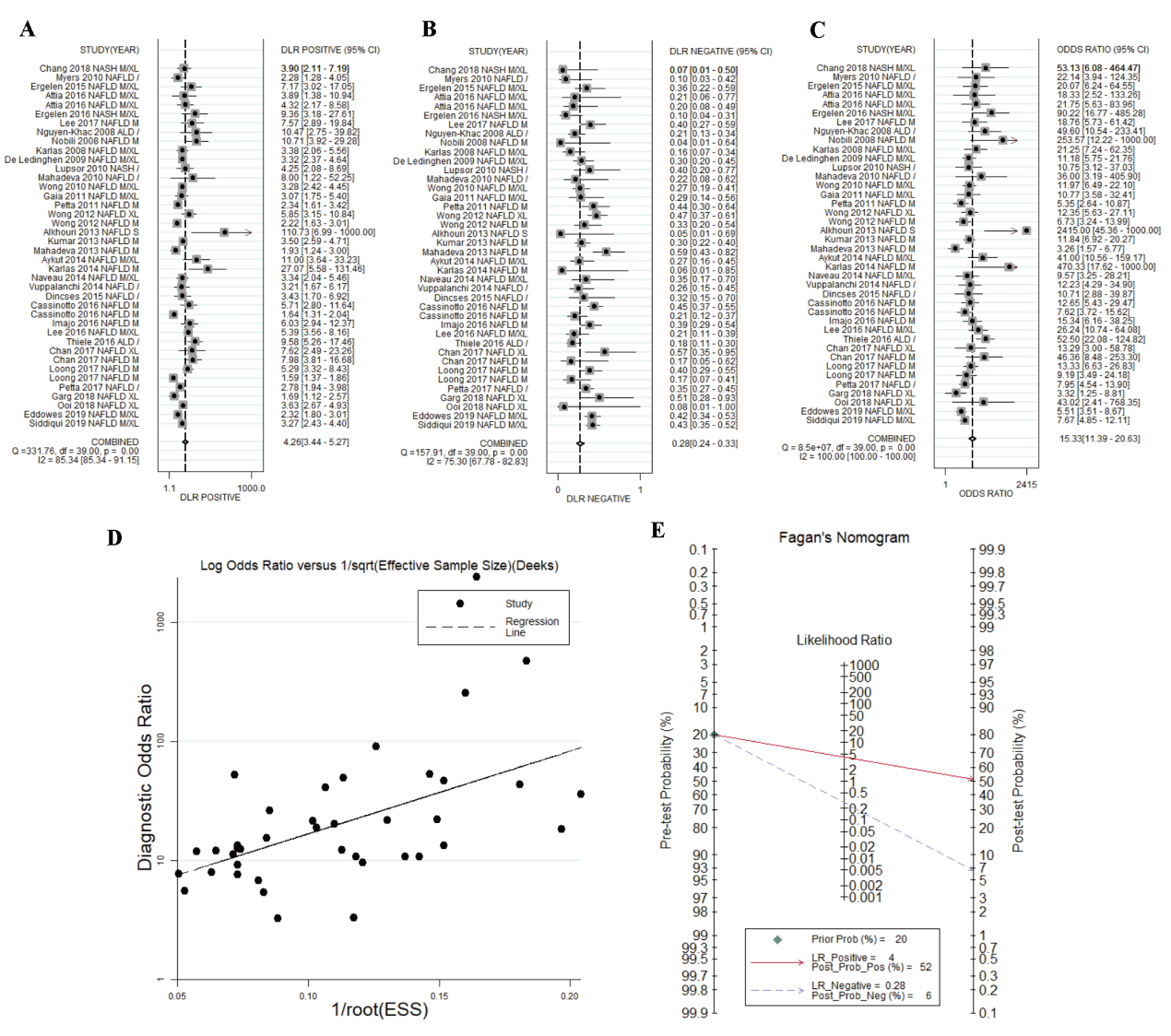
**

**Figure S6** Diagnostic performance of LSM for fibrosis grade ≥ F2 in alcoholic liver disease/non-alcoholic fatty liver disease. A: Positive likelihood; B: Negative likelihood; C: Diagnostic odds ratio; D: Deeks’ funnel plots; E: Fagan’s Nomogram.

**
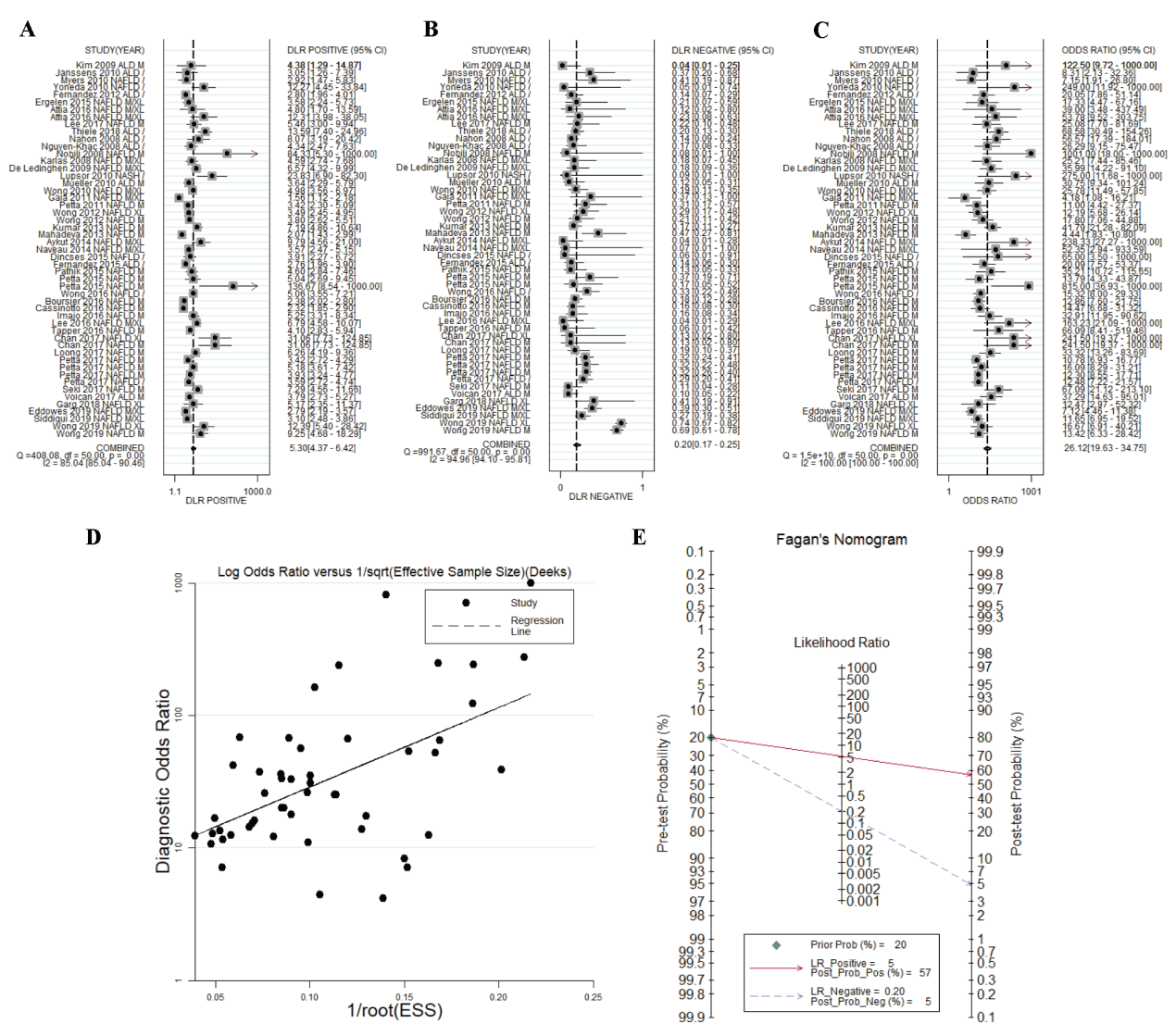
**

**Figure S7** Diagnostic performance of LSM for fibrosis grade ≥ F3 in alcoholic liver disease/non-alcoholic fatty liver disease. A: Positive likelihood; B: Negative likelihood; C: Diagnostic odds ratio; D: Deeks’ funnel plots; E: Fagan’s Nomogram.

**
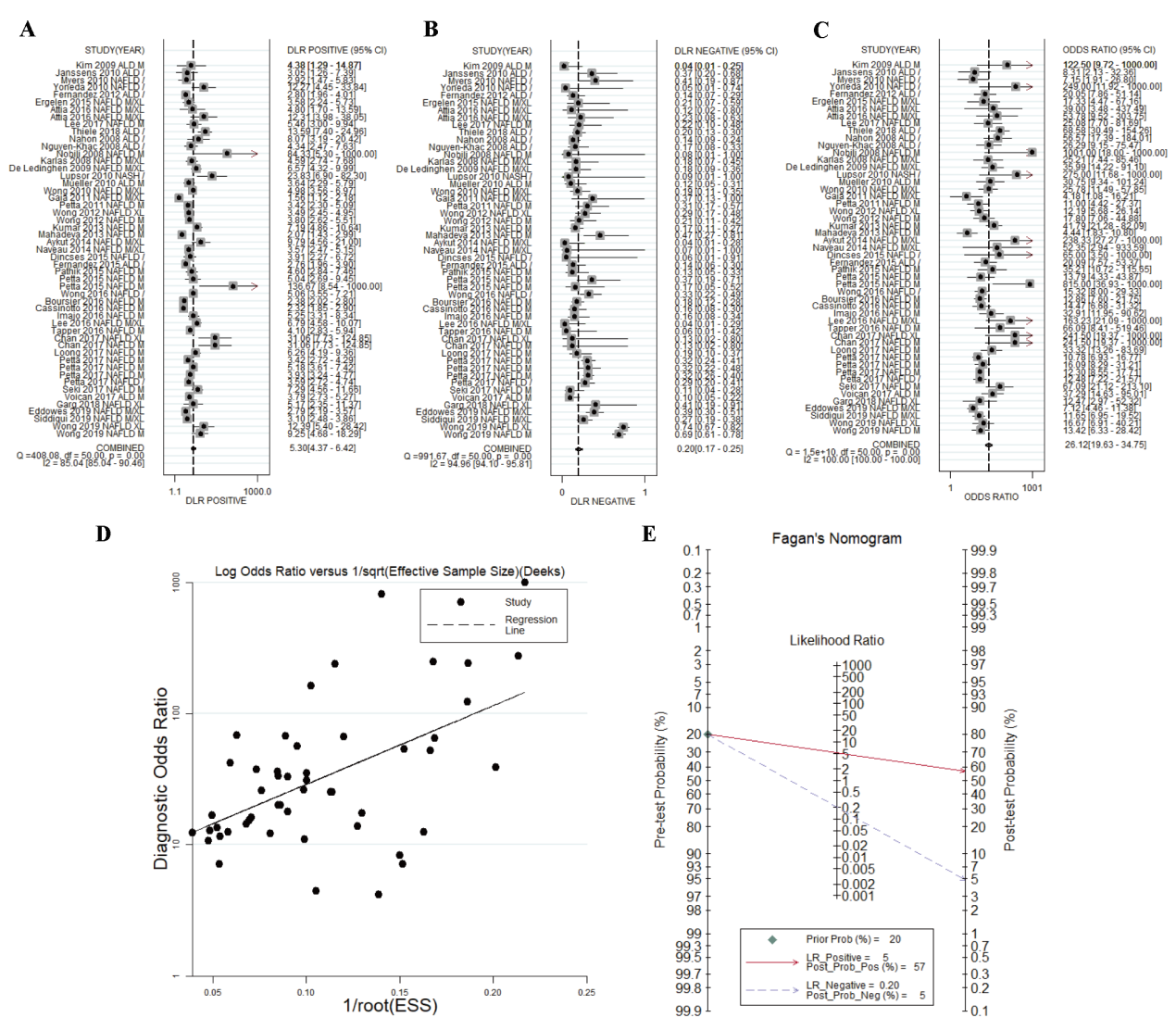
**

**Figure S8** Diagnostic performance of LSM for fibrosis grade = F4 in alcoholic liver disease/non-alcoholic fatty liver disease. A: Positive likelihood; B: Negative likelihood; C: Diagnostic odds ratio; D: Deeks’ funnel plots; E: Fagan’s Nomogram.

**References**

1 Wong VW-S, Irles M, Wong GL-H et al (2019) Unified interpretation of liver stiffness measurement by M and XL probes in non-alcoholic fatty liver disease. Gut. 10.1136/gutjnl-2018-317334

2 Siddiqui MS, Vuppalanchi R, Van Natta ML et al (2019) Vibration-Controlled Transient Elastography to Assess Fibrosis and Steatosis in Patients With Nonalcoholic Fatty Liver Disease. Clinical Gastroenterology and Hepatology 17:156-+

3 Eddowes PJ, Sasso M, Allison M et al (2019) Accuracy of FibroScan Controlled Attenuation Parameter and Liver Stiffness Measurement in Assessing Steatosis and Fibrosis in Patients With Non-alcoholic Fatty Liver Disease. Gastroenterology. 10.1053/j.gastro.2019.01.042

4 Runge JH, Smits LP, Verheij J et al (2018) MR Spectroscopy-derived Proton Density Fat Fraction Is Superior to Controlled Attenuation Parameter for Detecting and Grading Hepatic Steatosis. Radiology 286:547-556

5 Ooi GJ, Earnest A, Kemp WW et al (2018) Evaluating feasibility and accuracy of non-invasive tests for nonalcoholic fatty liver disease in severe and morbid obesity. Int J Obes (Lond) 42:1900-1911

6 Garg H, Aggarwal S, Shalimar et al (2018) Utility of transient elastography (fibroscan) and impact of bariatric surgery on nonalcoholic fatty liver disease (NAFLD) in morbidly obese patients. Surgery for Obesity and Related Diseases 14:81-92

7 Voican CS, Louvet A, Trabut J-B et al (2017) Transient elastography alone and in combination with FibroTest((R)) for the diagnosis of hepatic fibrosis in alcoholic liver disease. Liver International 37:1697-1705

8 Seki K, Shima T, Oya H, Mitsumoto Y, Mizuno M, Okanoue T (2017) Assessment of transient elastography in Japanese patients with non-alcoholic fatty liver disease. Hepatol Res 47:882-889

9 Petta S, Wong VW-S, Camma C et al (2017) Improved Noninvasive Prediction of Liver Fibrosis by Liver Stiffness Measurement in Patients With Nonalcoholic Fatty Liver Disease Accounting for Controlled Attenuation Parameter Values. Hepatology 65:1145-1155

10 Petta S, Wong VWS, Camma C et al (2017) Serial combination of non-invasive tools improves the diagnostic accuracy of severe liver fibrosis in patients with NAFLD. Alimentary Pharmacology & Therapeutics 46:617-627

11 Loong TC, Wei JL, Leung JC et al (2017) Application of the combined FibroMeter vibration-controlled transient elastography algorithm in Chinese patients with non-alcoholic fatty liver disease. J Gastroenterol Hepatol 32:1363-1369

12 Forlano R, Maurice J, Mullish BH et al (2017) The severity of steatosis does not influence liver stiffness measurements in patients with Non-Alcoholic Fatty Liver Disease. Journal of Hepatology 66:S586-S587

13 Chan WK, Mustapha NRN, Wong GLH, Wong VWS, Mahadeva S (2017) Controlled attenuation parameter using the FibroScan (R) XL probe for quantification of hepatic steatosis for non-alcoholic fatty liver disease in an Asian population. United European Gastroenterology Journal 5:76-85

14 Thiele M, Detlefsen S, Sevelsted Moller L et al (2016) Transient and 2-Dimensional Shear-Wave Elastography Provide Comparable Assessment of Alcoholic Liver Fibrosis and Cirrhosis. Gastroenterology 150:123-133

15 Tapper EB, Challies T, Nasser I, Afdhal NH, Lai M (2016) The Performance of Vibration Controlled Transient Elastography in a US Cohort of Patients With Nonalcoholic Fatty Liver Disease. Am J Gastroenterol 111:677-684

16 Mota M, Banini BA, Siddiqui M, Sanyal AJ (2016) Transient elastography use in the assessment of cirrhosis in patients with non-alcoholic steatohepatitis. American Journal of Gastroenterology 111:S449

17 Lee HW, Park SY, Kim SU et al (2016) Discrimination of Nonalcoholic Steatohepatitis Using Transient Elastography in Patients with Nonalcoholic Fatty Liver Disease. PLoS ONE 11:e0157358

18 Imajo K, Kessoku T, Honda Y et al (2016) Magnetic Resonance Imaging More Accurately Classifies Steatosis and Fibrosis in Patients With Nonalcoholic Fatty Liver Disease Than Transient Elastography. Gastroenterology 150:626-637.e627

19 Cassinotto C, Boursier J, de Ledinghen V et al (2016) Liver stiffness in nonalcoholic fatty liver disease: A comparison of supersonic shear imaging, FibroScan, and ARFI with liver biopsy. Hepatology 63:1817-1827

20 Boursier J, Vergniol J, Guillet A et al (2016) Diagnostic accuracy and prognostic significance of blood fibrosis tests and liver stiffness measurement by FibroScan in non-alcoholic fatty liver disease. Journal of Hepatology 65:570-578

21 Wong VW, Wong GLH, Shu S et al (2015) Application of transient elastography and FibroMeter in patients with nonalcoholic fatty liver disease. Hepatology 62:332A

22 Petta S, Vanni E, Bugianesi E et al (2015) The combination of liver stiffness measurement and NAFLD fibrosis score improves the noninvasive diagnostic accuracy for severe liver fibrosis in patients with nonalcoholic fatty liver disease. Liver International 35:1566-1573

23 Pathik P, Ravindra S, Ajay C, Prasad B, Jatin P, Prabha S (2015) Fibroscan versus simple noninvasive screening tools in predicting fibrosis in high-risk nonalcoholic fatty liver disease patients from Western India. Annals of Gastroenterology 28:281-286

24 Hiriart JB, Wong G, Vergniol J et al (2015) 42- Controlled Attenuation parameter (CAP) for the diagnosis of steatosis in nonalcoholic fatty liver disease. Hepatology 62:1271A

25 Fernandez M, Trepo E, Degre D et al (2015) Transient elastography using Fibroscan is the most reliable noninvasive method for the diagnosis of advanced fibrosis and cirrhosis in alcoholic liver disease. Eur J Gastroenterol Hepatol 27:1074-1079

26 Dincses E, Yilmaz Y (2015) Diagnostic usefulness of FibroMeter VCTE for hepatic fibrosis in patients with nonalcoholic fatty liver disease. Eur J Gastroenterol Hepatol 27:1149-1153

27 Vuppalanchi R, Gawrieh S, Weber R, Chalasani NP (2014) Is liver stiffness measurement (LSM) by Fibroscan better than FIB4 score for prediction of clinically significant fibrosis in patients with NAFLD? Hepatology 60:606A

28 Naveau S, Lamouri K, Pourcher G et al (2014) The diagnostic accuracy of transient elastography for the diagnosis of liver fibrosis in bariatric surgery candidates with suspected NAFLD. Obes Surg 24:1693-1701

29 Karlas T, Petroff D, Garnov N et al (2014) Non-invasive assessment of hepatic steatosis in patients with NAFLD using controlled attenuation parameter and 1H-MR spectroscopy. PLoS ONE 9:e91987

30 Chan WK, Nik Mustapha NR, Mahadeva S (2014) Controlled attenuation parameter for the detection and quantification of hepatic steatosis in nonalcoholic fatty liver disease. J Gastroenterol Hepatol 29:1470-1476

31 Aykut UE, Akyuz U, Yesil A et al (2014) A comparison of FibroMeter (TM) NAFLD Score, NAFLD fibrosis score, and transient elastography as noninvasive diagnostic tools for hepatic fibrosis in patients with biopsy-proven non-alcoholic fatty liver disease. Scand J Gastroenterol 49:1343-1348

32 Mahadeva S, Mahfudz AS, Vijayanathan A, Goh KL, Kulenthran A, Cheah PL (2013) Performance of transient elastography (TE) and factors associated with discordance in non-alcoholic fatty liver disease. J Dig Dis 14:604-610

33 Kumar R, Rastogi A, Sharma MK et al (2013) Liver Stiffness Measurements in Patients with Different Stages of Nonalcoholic Fatty Liver Disease: Diagnostic Performance and Clinicopathological Correlation. Digestive Diseases and Sciences 58:265-274

34 Alkhouri N, Sedki E, Alisi A et al (2013) Combined paediatric NAFLD fibrosis index and transient elastography to predict clinically significant fibrosis in children with fatty liver disease. Liver Int 33:79-85

35 Wong VW-S, Vergniol J, Wong GL-H et al (2012) Liver Stiffness Measurement Using XL Probe in Patients With Nonalcoholic Fatty Liver Disease. American Journal of Gastroenterology 107:1862-1871

36 Friedrich-Rust M, Romen D, Vermehren J et al (2012) Acoustic radiation force impulse-imaging and transient elastography for non-invasive assessment of liver fibrosis and steatosis in NAFLD. Eur J Radiol 81:e325-331

37 Petta S, Di Marco V, Camma C, Butera G, Cabibi D, Craxi A (2011) Reliability of liver stiffness measurement in non-alcoholic fatty liver disease: the effects of body mass index. Aliment Pharmacol Ther 33:1350-1360

38 Gaia S, Carenzi S, Barilli AL et al (2011) Reliability of transient elastography for the detection of fibrosis in non-alcoholic fatty liver disease and chronic viral hepatitis. J Hepatol 54:64-71

39 Wong VW, Vergniol J, Wong GL et al (2010) Diagnosis of fibrosis and cirrhosis using liver stiffness measurement in nonalcoholic fatty liver disease. Hepatology 51:454-462

40 Mueller S, Millonig G, Sarovska L et al (2010) Increased liver stiffness in alcoholic liver disease: Differentiating fibrosis from steatohepatitis. World J Gastroenterol 16:966-972

41 Mahadeva S, Mahfudz A, Vijayanathan A, Goh KL, Arumugam K, Cheah PL (2010) Accuracy of liver stiffness measurement in an Asian population with non-alcoholic fatty liver diseasea preliminary report. Journal of Gastroenterology and Hepatology 25:A102

42 Lupsor M, Badea R, Stefanescu H et al (2010) Performance of unidimensional transient elastography in staging non-alcoholic steatohepatitis. J Gastrointestin Liver Dis 19:53-60

43 De Ledinghen V, Wong VW, Vergniol J et al (2009) **92**--Prediction of fibrosis in patients with NAFLD using transient elastography : A prospective multicentre study**---**. Hepatology 50:767A-768A

44 Yoneda M, Yoneda M, Mawatari H et al (2008) **68--**Noninvasive assessment of liver fibrosis by measurement of stiffness in patients with nonalcoholic fatty liver disease (NAFLD). Dig Liver Dis 40:371-378

45 Nobili V, Vizzutti F, Arena U et al (2008) **69-**-Accuracy and reproducibility of transient elastography for the diagnosis of fibrosis in pediatric nonalcoholic steatohepatitis. Hepatology 48:442-448

46 Nguyen-Khac E, Chatelain D, Tramier B et al (2008) **70--**Assessment of asymptomatic liver fibrosis in alcoholic patients using fibroscan: prospective comparison with seven non-invasive laboratory tests. Alimentary Pharmacology & Therapeutics 28:1188-1198

47 Nahon P, Kettaneh A, Tengher-Barna I et al (2008) **71--**Assessment of liver fibrosis using transient elastography in patients with alcoholic liver disease. Journal of Hepatology 49:1062-1068

48 Thiele M, Rausch V, Fluhr G et al (2018) **72-**Controlled attenuation parameter and alcoholic hepatic steatosis: Diagnostic accuracy and role of alcohol detoxification. Journal of Hepatology 68:1025-1032

49 Naveau S, Voican CS, Lebrun A et al (2017) **73--**Controlled attenuation parameter for diagnosing steatosis in bariatric surgery candidates with suspected nonalcoholic fatty liver disease. Eur J Gastroenterol Hepatol 29:1022-1030

50 Lee MS, Bae JM, Joo SK et al (2017) **74--**Prospective comparison among transient elastography, supersonic shear imaging, and ARFI imaging for predicting fibrosis in nonalcoholic fatty liver disease. PLoS ONE 12:e0188321

51 Ergelen R, Yilmaz Y, Asedov R et al (2016) **75--**Comparison of Doppler ultrasound and transient elastography in the diagnosis of significant fibrosis in patients with nonalcoholic steatohepatitis. Abdom Radiol (NY) 41:1505-1510

52 de Ledinghen V, Wong GL, Vergniol J et al (2016) **76-**-Controlled attenuation parameter for the diagnosis of steatosis in non-alcoholic fatty liver disease. J Gastroenterol Hepatol 31:848-855

53 Attia D, Bantel H, Lenzen H, Manns MP, Gebel MJ, Potthoff A (2016) **77--Liver stiffness measurement using acoustic radiation force impulse** elastography in overweight and obese patients. Alimentary Pharmacology & Therapeutics 44:366-379

54 Ergelen R, Akyuz U, Aydin Y, Eren F, Yilmaz Y (2015) **79--**Measurements of serum procollagen-III peptide and M30 do not improve the diagnostic accuracy of transient elastography for the detection of hepatic fibrosis in patients with nonalcoholic fatty liver disease. Eur J Gastroenterol Hepatol 27:667-671

55 Kumar M, Rastogi A, Singh T et al (2013) **80--**Controlled attenuation parameter for non-invasive assessment of hepatic steatosis: does etiology affect performance? J Gastroenterol Hepatol 28:1194-1201

56 Fernandez M, Trepo E, Gustot T et al (2012) **84--**Fibroscan (Transient Elastography) is the most reliable non-invasive method for the assessment of severe fibrosis and cirrhosis in alcoholic liver disease. Hepatology 56:821A-822A

57 Yoneda M, Suzuki K, Kato S et al (2010) **87--**Nonalcoholic Fatty Liver Disease: US-based Acoustic Radiation Force Impulse Elastography. Radiology 256:640-647

58 Myers RP, Elkashab M, Ma M, Crotty P, Pomier-Layrargues G (2010) **88--**Transient elastography for the noninvasive assessment of liver fibrosis: a multicentre Canadian study. Can J Gastroenterol 24:661-670

59 Janssens F, De Suray N, Piessevaux H, Horsmans Y, De Timary P, Stärkel P (2010) **89--**Can transient elastography replace liver histology for determination of advanced fibrosis in alcoholic patients: A real-life study. J Clin Gastroenterol 44:575-582

60 Kim SG, Kim YS, Jung SW et al (2009) **91--**The usefulness of transient elastography to diagnose cirrhosis in patients with alcoholic liver disease]. Korean J Hepatol 15:42-51

61 Lemoine M, Katsahian S, Ziol M et al (2008) **94--**Liver stiffness measurement as a predictive tool of clinically significant portal hypertension in patients with compensated hepatitis C virus or alcohol-related cirrhosis. Alimentary Pharmacology & Therapeutics 28:1102-1110

62 Chang PE, Hartono JL, Ngai YL, Dan YY, Lim KB, Chow WC (2018) **96-**Optimal liver stiffness measurement values for the diagnosis of significant fibrosis and cirrhosis in chronic liver disease in Singapore. Singapore Med J. 10.11622/smedj.2018156
